# Effects of Intrinsic Foot Muscle-Targeted Exercise Programs on Functional, Neuromuscular, and Biomechanical Outcomes Relevant to Lower-Limb Injury: A Systematic Review with Limited Meta-Analysis

**DOI:** 10.3390/sports14100455

**Published:** 2026-10-14

**Authors:** Estêvão Rios Monteiro, Geovanna Trajano, Maria Luisa Pereira Castro, Leandro Alberto Calazans Nogueira, Victor Gonçalves Corrêa Neto, Francine De Oliveira, Alexandre Gonçalves de Meirelles, Jefferson da Silva Novaes

**Affiliations:** 1 Graduate Program in Rehabilitation Science, Centro Universitário Augusto Motta (UNISUAM), Rio de Janeiro 21032-060, Brazil; leandronogueira@souunisuam.com.br (L.A.C.N.); alexandremeirelles@souunisuam.com.br (A.G.d.M.); 2 Hospital Universitário dos Servidores do Estado (HUSE-UNIRIO), Federal University of the State of Rio de Janeiro, Rio de Janeiro 20221-161, Brazil; 3 Physiotherapy Department, Estácio de Sá University, Rio de Janeiro 21351-130, Brazil; 4 Undergraduate in Physical Therapy, Centro Universitário Augusto Motta, Rio de Janeiro 21032-060, Brazil; geovannatrajano@souunisuam.com.br (G.T.); marialuisacastro@souunisuam.com.br (M.L.P.C.); 5 Physiotherapy Department, Federal Institute of Rio de Janeiro (IFRJ), Rio de Janeiro 21715-000, Brazil; 6 School of Physical Education and Sports, Universidade Federal do Rio de Janeiro, Rio de Janeiro 21941-599, Brazil; victorgcn@hotmail.com (V.G.C.N.); francinerdeoliveiras@gmail.com (F.D.O.); 7 Strength Training Laboratory (LABFOR), Universidade Federal de Juiz de Fora, Juiz de Fora 36036-900, Brazil; jeffsnovaes@gmail.com; 8 Graduate Program in Physical Education, Universidade Federal da Paraiba, João Pessoa 58051-900, Brazil

**Keywords:** ankle injuries, exercise therapy, intrinsic foot muscles, knee injuries, rehabilitation

## Abstract

This review evaluated the effects of intrinsic foot muscle (IFM)-targeted exercise programs on predefined ankle- and knee-related clinical endpoints and on functional, neuromuscular, and biomechanical surrogate outcomes potentially relevant to lower-limb injury. This systematic review, incorporating a limited meta-analysis where quantitative pooling was feasible, followed PRISMA guidelines. Six electronic databases were searched for randomized controlled trials investigating IFM-targeted exercise programs in adults aged 18–59 years compared with no intervention, sham, or usual care, including parallel-group, cluster-randomized, and randomized crossover or counterbalanced designs when an adequate washout period was reported. Meta-analysis used Hedges’ g under a random-effects model with restricted maximum likelihood (REML) estimation of between-study variance and Hartung–Knapp adjustment for the pooled confidence interval; outcomes with fewer than three poolable studies were synthesized narratively per SWiM guidance. Thirteen randomized controlled trial reports (462 participants in the analyzed samples; 2012–2026) were included. The included samples were predominantly young and physically active, limiting generalizability across the broader adult age range. None of the included trials reported the predefined primary clinical endpoint categories: incident ankle injuries, prospectively recorded ankle-instability events, incident acute knee injuries, or incident knee-related symptoms/clinical syndromes. Consequently, none of these primary endpoint categories could be evaluated quantitatively or through comparative narrative synthesis, resulting in an empty synthesis for the primary clinical outcomes. Therefore, no direct estimate of the effect of IFM-targeted exercise programs on incident ankle injury, ankle-instability events, acute knee injury, or the onset of knee-related symptoms/clinical syndromes could be generated. The available evidence was therefore limited to secondary surrogate outcomes. Ten secondary outcome clusters were identified; however, quantitative pooling was feasible for only one dynamic postural control (k = 3; *n* = 70), while the remaining outcomes were synthesized narratively. For dynamic postural control, the pooled point estimate favored IFM training in direction (Hedges’ g = 0.39), but the effect was highly uncertain under the REML/Hartung–Knapp model (95% CI −0.62 to 1.41; *p* = 0.238), and the certainty of evidence was rated Very Low. Individual trials reported improvements in foot muscle strength, morphology, foot posture, ankle instability, and inter-articular coordination, but these findings were generally derived from single studies and cannot establish injury-preventive efficacy. The evidence base is currently empty for the predefined primary outcomes, and the current evidence does not support clinical recommendations for isolated IFM training as an injury-prevention strategy or as an intervention with established broader rehabilitation effectiveness. PROSPERO registration: CRD420261405154.

## 1. Introduction

Lower-limb musculoskeletal injuries represent a major source of morbidity, functional impairment, and socioeconomic burden in adults. Lateral ankle sprain is the most prevalent joint injury, with a pooled incidence of approximately 11.55 cases per 1000 exposures, disproportionately affecting women and young adults [1]; in military populations, incidence exceeds 58 cases per 1000 person-years [2]. Anterior cruciate ligament rupture affects 68.6 per 100,000 person-years, imposing substantial surgical costs and long-term functional sequelae [3], while patellofemoral pain syndrome (PFPS) affects approximately 22.7% of the general population, with greater prevalence in women and significant interference with daily activities [4]. Collectively, these conditions underscore the clinical and scientific urgency of identifying modifiable mechanisms amenable to prevention and rehabilitation.

The lower-limb kinetic chain originates at foot–ground contact, and disruptions in distal neuromechanical control may propagate proximally, compromising ankle, knee, hip, and trunk function. McKeon et al. [5] formalized this concept through the foot core paradigm, conceptualizing the foot as an integrated system of passive structures (bones, plantar fascia, ligaments), active components (intrinsic and extrinsic muscles), and neural elements (plantar mechanoreceptors) directly analogous to the lumbopelvic core. Within this framework, the intrinsic foot muscles (IFMs), including the flexor digitorum brevis, abductor hallucis, quadratus plantae, lumbricals, interossei, and flexor hallucis brevis, function as local stabilizers of the medial longitudinal arch, modulating subtalar pronation, midfoot stiffness, and proprioceptive afference underlying static and dynamic balance [5,6].

Despite this biomechanical relevance, IFMs have been systematically underrepresented in rehabilitation and prevention programs. Excessive pronation, commonly characterized using measures such as navicular drop and the Foot Posture Index, has been associated with select lower-limb conditions and may reflect aspects of distal biomechanical function. However, these measures should be considered intermediate or potentially injury-related markers rather than validated substitutes for prospectively measured injury incidence [4,7]. Individuals with CAI may demonstrate deficits in proprioception, dynamic balance, and intrinsic foot muscle activation, and some of these intermediate neuromuscular and functional measures appear modifiable through targeted IFM training [8,9]. Such changes may be mechanistically relevant but should not be interpreted as evidence of reduced injury incidence. Similarly, landing kinematics, tibial rotation, rearfoot motion, muscle morphology, muscle strength, and related biomechanical variables may contribute to mechanistic models of injury susceptibility, but they remain intermediate measures and cannot substitute for directly observed ankle or knee injury outcomes [7,10]. Accordingly, foot-core function represents a biologically plausible mechanistic target, while the extent to which changes in strength, morphology, balance, coordination, or foot posture translate into fewer clinically observed injuries remains uncertain.

Previous systematic reviews have established that IFM-targeted or foot-focused interventions can modify several foot-specific and functional outcomes, but their scope differs importantly from the clinical question addressed in the present review. Wei et al. [11] synthesized evidence primarily on foot function, medial longitudinal arch morphology, and dynamic postural balance, whereas Jaffri et al. [12] examined a broader set of foot-function outcomes, including foot posture, balance, strength, patient-reported outcomes, sensory function, and motor performance. More recently, Peters-Dickie et al. [13] synthesized randomized trials of both specific foot exercises and minimalist footwear, focusing on foot-muscle size, strength, and biomechanical outcomes. Collectively, these reviews demonstrate that foot-focused interventions may produce local neuromuscular and biomechanical adaptations; however, they were not designed to determine whether effects attributable specifically to isolated IFM training translate into prospectively observed ankle or knee clinical events.

Accordingly, the unresolved evidence gap is not whether foot-focused interventions can modify surrogate foot-related measures, but whether IFM-targeted exercise programs have been evaluated against clinically observed outcomes relevant to lower-limb injury. The present review therefore differs from previous syntheses by restricting its intervention scope to exercise programs primarily designed to target the intrinsic foot musculature, defining clinically distinct ankle- and knee-related endpoints a priori, separating these endpoints from biomechanical, neuromuscular, and functional surrogates, and updating the evidence base through June 2026. The review was specifically designed to determine whether the mechanistic and functional adaptations associated with IFM-targeted exercise programs have been accompanied by evidence of downstream clinical benefit.

The primary objective of this systematic review was therefore to determine whether IFM-targeted exercise programs affect predefined ankle- and knee-related clinical outcomes in adults. Secondary objectives were to synthesize their effects on injury-relevant functional, neuromuscular, and biomechanical surrogate outcomes, including postural control, foot muscle strength and morphology, foot posture, proprioception, and inter-articular coordination, and to descriptively characterize variation in exercise type, weekly dose, program duration, and participants’ clinical or biomechanical profile where reported, rather than to estimate formal moderator effects.

## 2. Materials and Methods

This systematic review, with limited meta-analysis where quantitative pooling was feasible, followed the Preferred Reporting Items for Systematic Reviews and Meta-Analyses (PRISMA) guidelines [14] and was registered in the PROSPERO platform (CRD420261405154) on 4 June 2026. Because the archived materials available for this revision do not independently verify that formal screening commenced only after registration, the review is not described as prospectively registered. Formal title/abstract screening commenced on 20 May 2026. The eligibility framework, predefined primary and secondary outcomes, and planned synthesis framework had been established before data extraction, and the predefined primary outcomes were not modified in response to the observed results. The original PROSPERO record (version 1.0, published 4 June 2026) explicitly documented the intervention-dose eligibility thresholds of at least 4 weeks and at least three weekly training sessions, or an equivalent reported dose. Because registration occurred after title/abstract screening had commenced, these numerical thresholds are reported as prespecified in the registered record, but not as prospectively registered before screening.

### 2.1. Identification of Relevant Studies

Studies were retrieved through electronic database searches and a comprehensive scan of the reference lists of included studies. Preliminary database searches were conducted in May 2026, and formal title/abstract screening commenced on 20 May 2026 using the database-specific exports available at that stage. Because exact preliminary run dates were not retained consistently for every database, these initial searches are reported as occurring in May 2026 rather than being assigned retrospectively reconstructed dates. To standardize the coverage date across sources and establish the final reproducible search set, all six databases included in the review were rerun on 5 June 2026: PubMed/MEDLINE (5 June 2026), the Cochrane Central Register of Controlled Trials (CENTRAL) (5 June 2026), PEDro (5 June 2026), Web of Science (5 June 2026), LILACS (5 June 2026), and SciELO (5 June 2026). The 5 June 2026 reruns constituted the final search update and generated the database-specific record counts reported in the PRISMA flow diagram and the Results section; preliminary outputs were not counted as additional independent records. Each database was searched from inception to the search date without publication-date restrictions. The archived search materials did not indicate a change in the conceptual search framework during screening; differences among database-specific strategies reflected only the controlled vocabulary, field codes, Boolean syntax, and interface requirements of each source. The BVS export contained records from multiple constituent databases; therefore, only records explicitly indexed as LILACS (n = 150) were retained for the LILACS-specific dataset to avoid counting MEDLINE and other BVS sources as independent databases. The search strategy combined descriptors and Boolean operators (AND/OR/NOT) for each database and is available as Supplementary Materials (https://doi.org/10.17605/OSF.IO/TSMQG). The database-specific yields were verified against the archived search outputs. Zero records retrieved from Cochrane CENTRAL and PEDro represent the actual results of the executed database-specific searches and do not indicate that these sources were omitted or incompletely searched. No additional formal database search is documented after the 5 June 2026 final update.

### 2.2. Eligibility Criteria

Studies were included according to Population, Intervention, Comparator, Outcome, and Study Design (PICOS) criteria [15]: (P) comprised adults from the general population, aged ≥18 years, of both sexes. This included asymptomatic individuals, individuals at increased risk due to biomechanical characteristics (e.g., pronated foot, increased navicular drop, functional instability without recent surgery), and individuals with a clinical diagnosis of chronic ankle instability or patellofemoral pain syndrome. Exclusion criteria included lower-limb orthopedic surgery within the previous 12 months; central or peripheral neurological diseases; active inflammatory rheumatic diseases; amputation; diabetes with documented peripheral neuropathy; and pregnancy. (I) comprised intrinsic foot muscle (IFM)-targeted exercise programs, either supervised or home-based, including programs centered on exercises such as the short-foot exercise (SFE), toe curl/towel curl, toe spread, doming, hallux exercises, and toe exercises involving metatarsophalangeal flexion.

Eligible interventions were required to last ≥4 weeks and to include at least three training sessions per week or an equivalent reported dose. These numerical thresholds were explicitly specified in the original PROSPERO record (CRD420261405154, version 1.0). They were adopted as operational criteria to distinguish sustained IFM training programs from acute, single-session, or short-term familiarization exposures and to ensure that the intervention represented a repeated training stimulus rather than a transient exposure. The thresholds were not modified in response to the direction or magnitude of the retrieved study results. Intervention eligibility was operationalized at the level of the analyzed study arm. An eligible arm had to be primarily designed to target the intrinsic foot musculature and could include ancillary foot- or ankle-focused exercises when these were integrated within the same IFM-targeted program rather than delivered as a distinct concurrent co-intervention. Programs were excluded when the analyzed arm combined IFM training with an independent lower-limb strengthening, footwear, neurostimulation, or other active component that prevented a clinically interpretable IFM-targeted contrast. Accordingly, ancillary exercises incorporated within an IFM-targeted package were considered part of the intervention package and were documented descriptively; effects from such trials were interpreted as effects of the IFM-targeted program rather than as effects attributable exclusively to intrinsic-foot-muscle contraction. Isolated plantar orthoses, minimalist footwear alone, isolated electrical stimulation, and isolated manual therapy were excluded. For (C), eligible comparators included no intervention, wait-list control, sham intervention (e.g., tactile contact without specific muscle activation), usual care, isolated stretching, or educational instruction without strength training. To be eligible, a study was required to include at least one eligible IFM-targeted intervention arm and at least one comparator arm that did not provide a competing structured lower-limb exercise, neuromuscular, or footwear-based intervention expected to influence the same outcomes. Studies in which the only available comparator consisted of another structured lower-limb exercise or strengthening program, a neuromuscular intervention, or a footwear-based intervention were excluded because they did not provide an eligible control condition for estimating the effect of the IFM-targeted program. In multi-arm trials that included both an eligible control condition and one or more additional active intervention arms, the study remained eligible; however, only contrasts between the eligible IFM-targeted arm and the eligible control were used in the quantitative or structured narrative synthesis. Additional active arms were retained for descriptive characterization but were not treated as control comparators for the primary effect synthesis. Regarding (O), primary outcomes were (1) ankle-related clinical outcomes, prespecified as two conceptually distinct endpoint categories: (a) incident ankle injuries, including first or recurrent lateral ankle sprains, and (b) prospectively recorded ankle-instability events, including episodes of giving way.

Giving-way episodes were treated as instability events rather than new injuries because they may occur without a discrete traumatic injury event. These ankle endpoint categories were to be analyzed and interpreted separately and were not considered interchangeable measures of injury incidence. Another was (2) knee-related clinical outcomes, prespecified as two conceptually distinct endpoint categories: (a) incident acute knee injuries, defined as prospectively observed new injury events, and (b) incident or newly developed knee-related symptoms/clinical syndromes, including patellofemoral pain syndrome, patellar tendinopathy, and iliotibial band-related symptoms. These knee endpoint types were analyzed and interpreted separately because symptom or syndrome onset is not clinically equivalent to an acute injury event. Secondary outcomes included dynamic balance/postural control, assessed using reach-based tests such as the Star Excursion Balance Test (SEBT) or Y-Balance Test (YBT) and, where reported, instrumented center-of-pressure (COP) measures obtained during a dynamic-balance task; static balance/postural control, assessed using COP measures obtained during quiet or static stance/posturography; self-reported function, including the Foot and Ankle Ability Measure (FAAM), Cumberland Ankle Instability Tool (CAIT), Kujala Anterior Knee Pain Scale, and Knee injury and Osteoarthritis Outcome Score (KOOS); IFM strength, assessed by paper grip test or hallux dynamometry; foot biomechanics, including navicular drop, Foot Posture Index (FPI-6), and arch height index; pain, assessed by the visual analog scale (VAS); and adverse events. (S) comprised randomized controlled trials, including parallel-group, cluster-randomized, and randomized crossover or counterbalanced designs when an adequate washout period was reported. Non-randomized controlled studies, quasi-experimental studies, cross-sectional studies, case series, case reports, observational studies without intervention, protocols without outcome data, dissertations, theses, conference abstracts, and other non-peer-reviewed or non-indexed grey literature were excluded.

Studies were eligible if they: (1) were original peer-reviewed reports published without temporal restrictions; (2) included an analyzed intervention arm primarily designed to target the intrinsic foot musculature; and (3) used a randomized controlled design, including parallel-group, cluster-randomized, and randomized crossover or counterbalanced designs when an adequate washout period was reported. Ancillary foot- or ankle-focused exercises incorporated within the same IFM-targeted intervention package were permitted when they were not separable as an independent concurrent treatment. Accordingly, such trials were interpreted as evaluating the overall IFM-targeted exercise program rather than the isolated physiological effect of intrinsic-foot-muscle contraction alone. Studies were excluded if they: (1) were not published in English or Portuguese; (2) combined the IFM-targeted intervention with a distinct active co-intervention that prevented attribution of the study contrast to the IFM-targeted program (e.g., separate hip strengthening, neurostimulation, or footwear intervention); (3) used non-randomized, quasi-experimental, observational, or other ineligible designs; (4) were theses, dissertations, conference abstracts, protocols without outcome data, or other grey literature; or (5) involved animal models.

The restriction to English- and Portuguese-language reports was pragmatic and reflected the linguistic expertise and translation resources available to the review team. Records published in other languages were excluded during study selection when a reliable translation was not available to support accurate eligibility assessment and data extraction. This restriction may have reduced the comprehensiveness of the evidence base and was therefore considered a potential source of language bias.

Following refinement of this operational intervention definition, all reports undergoing full-text eligibility assessment were reassessed against the same criteria to ensure consistent application across studies. This reassessment did not alter the final set of included studies. All 13 included trials contained at least one analyzed intervention arm primarily designed to target the intrinsic foot musculature; ancillary foot- or ankle-focused exercises were permitted only when they were integrated within the same IFM-targeted exercise program and were not separable as an independent concurrent co-intervention.

### 2.3. Study Selection and Data Extraction

Records retrieved from each database were imported into Rayyan software (version 1.7.8, Cambridge, MA, USA) [16] and screened in database-specific batches. Titles and abstracts were assessed according to the eligibility criteria by two independent researchers, with conflicts resolved by a third reviewer; reviewers were not blinded to authors, institutions, or journals. Because the archived workflow screened source-specific exports before cross-database reconciliation, duplicate checking across databases was performed after title/abstract screening among records retained for full-text assessment. Potentially eligible records were reconciled using bibliographic information (title, authors, year, and journal), and duplicate reports were retained only once before full-text retrieval. Abstracts lacking decisive information were selected for full-text inspection. A formal inter-reviewer agreement statistic was not calculated contemporaneously for screening. Because the archived screening records do not preserve the complete paired pre-consensus decisions for both reviewers across all screened records, a valid retrospective calculation of Cohen’s κ was not possible; agreement was therefore managed through the predefined independent dual-review process and adjudication procedure described above.

For each included study, the following information was extracted: bibliographic characteristics; sample size; participant characteristics, including age, sex, anthropometric characteristics, and training or clinical status where reported; intervention and comparator characteristics; exercise type, frequency, duration, and delivery format; outcome definitions and measurement instruments; assessment time points; and numerical or descriptive outcome data relevant to the predefined synthesis domains. For quantitative synthesis, post-intervention means and standard deviations were extracted when available, while baseline values were additionally reviewed to identify potentially important pre-intervention imbalances. Change-score data were considered only when sufficiently reported to permit analysis without unverifiable assumptions, as described in Section 2.5. For multi-arm trials, data extraction for the primary synthesis focused on the eligible IFM-targeted versus eligible control contrast, while additional active arms were retained only for descriptive characterization where relevant.

Randomized between-group contrasts between the eligible IFM-targeted arm and the eligible control condition were prioritized over within-group pre–post changes. Between-group effect estimates and 95% confidence intervals were reported when they were available in the extracted primary-study data; unreported confidence intervals were not retrospectively reconstructed. Interpretive statements made by the primary-study authors were recorded separately from observed findings, and any proposed injury-prevention implications were treated as hypotheses rather than observed effects when injury incidence had not been prospectively measured.

### 2.4. Risk of Bias and Methodological Quality Assessment

Risk of bias was evaluated using complementary study-level descriptive and result-specific approaches. At the study level, the Cochrane Risk of Bias tool, version 1 (RoB 1) [17], was retained as a descriptive mapping of recurrent methodological and reporting features across all included randomized trials. Its retention was not based on the number or type of outcomes reported by the trials and should not be interpreted as implying that RoB 2 is unsuitable for trials with multiple outcomes. Rather, RoB 1 provided a common descriptive overview across the heterogeneous evidence base and was not used to generate an overall study-level risk-of-bias judgment, to replace result-specific RoB 2 assessments, or to determine GRADE certainty. The standard RoB 1 domains evaluated were random sequence generation, allocation concealment, blinding of participants and personnel, blinding of outcome assessors, incomplete outcome data, selective outcome reporting, and other bias. Exercise-specific concerns related to equipment, participant effort, and familiarization or learning effects that had been recorded during the study-level assessment were treated as supporting considerations within the single ‘other bias’ domain rather than as separate Cochrane domains. No algorithmic overall RoB 1 classification was assigned. Because RoB 2 is designed to evaluate risk of bias for a specific result, an outcome-specific RoB 2 assessment was additionally conducted for the three trials contributing to the only meta-analyzed outcome, dynamic postural control [18,19,20]. RoB 2 was applied to the effect of assignment to intervention and assessed five domains: (1) bias arising from the randomization process; (2) bias due to deviations from intended interventions; (3) bias due to missing outcome data; (4) bias in measurement of the outcome; and (5) bias in selection of the reported result.

Overall judgments were derived according to the RoB 2 algorithm and classified as low risk of bias, some concerns, or high risk of bias. Lack of participant or therapist blinding was not considered sufficient, by itself, to determine high risk of bias; judgments considered whether awareness of intervention assignment could plausibly lead to deviations from the intended intervention or influence the specific outcome being assessed. All risk-of-bias assessments were performed independently by two reviewers, with disagreements resolved through discussion and consensus and, when necessary, consultation with a third reviewer. Outcome-specific RoB 2 judgments were subsequently considered in the risk-of-bias domain of the GRADE assessment for dynamic postural control. A formal inter-reviewer agreement coefficient was not calculated contemporaneously for the risk-of-bias assessments. Complete paired pre-consensus domain-level judgments were not retained in the archived review materials; consequently, Cohen’s κ could not be estimated retrospectively without reconstructing reviewer decisions after consensus.

Methodological characteristics of the included trials were additionally described using the Physiotherapy Evidence Database (PEDro) scale [21], an 11-item instrument developed for randomized trials in physical therapy (inter-rater reliability: ICC = 0.68; 95% CI: 0.57–0.76) [22]. PEDro was used to summarize selected design and reporting features, including random allocation, allocation concealment, blinding, completeness of follow-up, intention-to-treat analysis, between-group comparisons, and reporting of point estimates and variability. PEDro scores were interpreted descriptively and were not used as a global measure of internal validity, as a substitute for the Cochrane risk-of-bias assessments, or as an inclusion or exclusion criterion. Thus, PEDro and Cochrane assessments were treated as complementary but conceptually distinct: PEDro summarized selected methodological and reporting characteristics, whereas the Cochrane tools were used to evaluate the potential for bias in intervention-effect estimates.

The certainty of evidence was assessed using the GRADE framework as described in the Cochrane Handbook [23], considering risk of bias, inconsistency, indirectness, imprecision, and publication bias. GRADE does not require statistical pooling and can be applied to bodies of evidence synthesized using methods other than meta-analysis. In the present review, a formal GRADE assessment was conducted for the dynamic postural-control domain because it was the only secondary outcome represented by a sufficiently coherent body of evidence to support an outcome-level certainty judgment. The remaining surrogate outcomes were represented by sparse and clinically heterogeneous evidence, generally involving one or two studies with different outcome definitions and measurement approaches; these outcomes were therefore synthesized according to SWiM guidance [24] without formal certainty ratings. The predefined primary clinical endpoint categories—incident ankle injuries, prospectively recorded ankle-instability events, incident acute knee injuries, and incident knee-related symptoms/clinical syndromes—were not assigned GRADE certainty ratings because no eligible trial reported any of these endpoint categories and, consequently, no body of evidence was available to rate. GRADE judgments were made across the five standard domains and are presented, together with explicit reasons for downgrading, in the Summary of Findings.

### 2.5. Data Synthesis and Statistical Analysis

The predefined primary clinical outcome framework comprised ankle-related and knee-related clinical outcomes. Within the ankle domain, incident ankle injuries (including first or recurrent lateral ankle sprains) and prospectively recorded giving-way episodes were treated as conceptually distinct endpoint categories. Giving-way episodes were classified as ankle-instability events rather than new injuries and were not combined analytically with incident sprains. Within the knee domain, incident acute knee injuries and incident or newly developed knee-related symptoms/clinical syndromes were likewise treated as conceptually distinct endpoint categories and were not combined analytically because they represented different clinical event types. If no eligible study reported a predefined primary endpoint category, this was classified as an empty synthesis for that endpoint, and no quantitative effect estimate was calculated. In such cases, the absence of direct evidence was reported explicitly before proceeding to synthesis of secondary biomechanical, neuromuscular, or functional surrogate outcomes. For multi-arm trials, only contrasts between an eligible IFM-targeted intervention arm and an eligible control condition were included in the primary synthesis. Additional active intervention arms were not pooled with eligible control conditions and were not used as comparators for the primary quantitative or structured narrative effect estimates.

Eligibility for quantitative pooling was determined primarily by clinical, methodological, and construct comparability rather than by study number alone. Studies were considered sufficiently comparable when the intervention contrast, participant characteristics, outcome construct, measurement context and timing, and available effect estimates were judged to support a clinically interpretable pooled estimate. Only after this comparability assessment was an additional conservative operational threshold of at least three studies (k ≥ 3) applied. This threshold was specific to the present review and was not intended as a universal requirement for meta-analysis. It was adopted to avoid emphasizing pooled estimates based on only two studies, for which the estimated average effect, between-study variance, and heterogeneity are particularly dependent on the individual contributing studies. Accordingly, k ≥ 3 was treated as a necessary but not sufficient condition for quantitative synthesis. Outcome clusters failing either the comparability assessment or the study-number threshold were synthesized using a structured narrative approach following SWiM guidance [24]. For outcome clusters synthesized without meta-analysis, studies were grouped according to the predefined outcome construct. Within each cluster, the synthesis described the number of contributing studies and participants, the direction of effects relative to the eligible control condition, the approximate magnitude of effects when sufficiently reported, consistency or inconsistency across studies, relevant methodological limitations informed by the descriptive RoB 1 assessment, and clinical or measurement factors limiting comparability. Statistical significance alone was not used to classify an effect as favorable or unfavorable, and no pseudo-pooled effect estimate was generated when sufficiently comparable estimates were unavailable. For the dynamic postural-control domain, the three contributing trials used related but non-interchangeable measures. Pooling was undertaken because the measures were judged to represent the same broader functional construct—the ability to maintain postural stability during a challenging balance task requiring active control of the body over a constrained or changing base of support—and because the intervention contrasts and assessment timing were considered sufficiently compatible.

Reach-based outcomes and dynamic-task center-of-pressure measures were therefore grouped only when they reflected dynamic balance performance rather than quiet-standing posturography. For Lynn et al. [20], the effect entered into the quantitative synthesis was specifically the mediolateral range of COP movement recorded during the dynamic-balance task; the separate COP measure obtained during the static-balance test was classified as static postural control and was not included in the meta-analysis. The use of standardized mean differences permitted comparison across different measurement scales but was not considered to eliminate differences in measurement properties or construct operationalization. The pooled estimate was therefore interpreted as a domain-level summary of dynamic postural-control performance rather than as an estimate for any individual balance test. Narrative synthesis was not considered, by itself, a reason to preclude GRADE assessment; rather, formal GRADE ratings were limited to outcome domains for which the available studies constituted a sufficiently coherent body of evidence for an interpretable certainty judgment. Given that only one outcome cluster met both the comparability and study-number criteria for quantitative pooling and that this meta-analysis included only three studies, formal moderator analyses (including subgroup analysis or meta-regression) were not considered statistically reliable. Exercise type, weekly dose, program duration, and participants’ clinical or biomechanical profiles were therefore summarized descriptively as study characteristics rather than analyzed as effect modifiers. Accordingly, the review objective was framed as descriptive characterization of these intervention and participant features, not as a formal moderator analysis.

When a trial reported more than one potentially eligible result within the same outcome domain, a deterministic within-study selection hierarchy was applied so that no more than one effect estimate from the same independent participant group contributed to a given meta-analysis. The result explicitly designated as primary by the original trial was prioritized when it matched the synthesis domain. If no primary result was identified, a composite or global score was preferred over individual components (e.g., an SEBT/YBT composite score over individual reach directions) when available. For limb-specific outcomes, the affected or symptomatic limb was prioritized in clinical populations and the specifically trained limb in unilateral interventions; otherwise, the test limb designated in the original report was used. For studies reporting more than one eligible post-intervention assessment, the measurement obtained immediately after completion of the intervention was used for the primary synthesis, with later retention or washout assessments treated separately. If multiple eligible measures remained after these criteria, the measure with the closest conceptual correspondence to the measures contributed by the other studies in the same outcome cluster was selected. Statistical significance, effect direction, and effect magnitude were not considered in this selection process. Because the archived protocol did not retain a sufficiently granular prospective hierarchy covering every possible combination of test direction, limb, time point, and balance metric, this rule is reported transparently as the deterministic hierarchy applied to the final synthesis rather than retrospectively described as prospectively prespecified.

For the pooled analysis, between-group standardized mean differences were calculated using Hedges’ g [25] to correct for small-sample bias. Post-intervention means and standard deviations were used as the primary input because all studies contributing to the quantitative synthesis were randomized controlled trials, and this approach preserves the randomized between-group comparison without requiring assumptions regarding the correlation between baseline and post-intervention measurements. Baseline values for the dynamic postural-control outcome were reviewed in the contributing trials, when reported, to identify potentially important pre-intervention imbalances between intervention and control groups. However, the primary reports did not provide sufficiently complete and homogeneous information to derive standardized baseline-adjusted estimates for all three trials. Because the primary effect measure was a standardized mean difference, post-intervention and change-score SMDs were not combined within the same pooled analysis, as the standard deviations used for standardization represent different sources of variability. A separate change-from-baseline sensitivity analysis was planned for studies reporting mean changes together with their corresponding standard deviations, or sufficient information to derive them without unverifiable assumptions. Where the standard deviation of change was not reported and could only be estimated by assuming an unknown pre–post correlation, no imputation was undertaken. Consequently, a pooled change-score sensitivity analysis across all three studies could not be performed robustly from the published data. Outcome direction was harmonized before standardization so that positive values consistently favored IFM training. For reach-based measures such as the SEBT or Y-Balance Test, greater reach distance was interpreted as better dynamic postural-control performance; where reach distances were normalized to limb length in the source study, normalized values were retained.

For the mediolateral COP-excursion measure contributed by Lynn et al. [20] during the dynamic-balance task, lower excursion was interpreted as better postural control and the sign of the effect estimate was therefore reversed. A random-effects model was fitted using restricted maximum likelihood (REML) to estimate the between-study variance (τ^2^) [23]. Because only three studies contributed to the quantitative synthesis, the Hartung–Knapp adjustment was applied to the confidence interval around the pooled effect to provide a more conservative estimate of uncertainty with a small number of studies. A 95% prediction interval was additionally calculated using the REML estimate of between-study variance and a t distribution with k−2 degrees of freedom to illustrate the range of true effects that might be expected in a future comparable study. Because only three studies were available, this prediction interval was interpreted as exploratory and with substantial caution, given the instability of between-study variance estimation when k is very small. Heterogeneity was quantified using Cochran’s Q (α = 0.10), the I^2^ statistic, and τ^2^, following recommended thresholds: I^2^ of 0–40% indicating low heterogeneity, 30–60% moderate, 50–90% substantial, and 75–100% considerable [26]. Given the very small number of pooled studies, Cochran’s Q and I^2^ were interpreted cautiously because both statistics have limited power and precision for detecting or quantifying between-study heterogeneity when k is small. Accordingly, a low observed I^2^ value was not considered evidence of clinical, methodological, or construct homogeneity. The DerSimonian–Laird estimator [27] was retained as a sensitivity analysis to evaluate whether the choice of between-study variance estimator materially altered the direction or statistical interpretation of the pooled effect. A post hoc population-and-measurement sensitivity analysis was also undertaken by excluding Lynn et al. [20], thereby removing both the healthy cohort and the only trial contributing a dynamic COP-excursion measure rather than a reach-distance measure, and restricting the comparison to the two trials enrolling participants with chronic ankle instability [18,19]. Because only two studies remained, this analysis was interpreted descriptively as a robustness check rather than as a definitive pooled estimate.

Publication bias was not formally assessed using funnel plot asymmetry or Egger’s regression test, as the number of studies per cluster (k < 10) was insufficient to support reliable estimation [22]. A leave-one-out sensitivity analysis was performed to evaluate the influence of individual studies on the pooled estimate. All analyses were conducted in R version 4.3 [28] using the meta package version 7.0 [29]; two-sided *p*-values < 0.05 were considered statistically significant (https://doi.org/10.17605/OSF.IO/TSMQG).

## 3. Results

### 3.1. Study Selection

**Figure 1:**
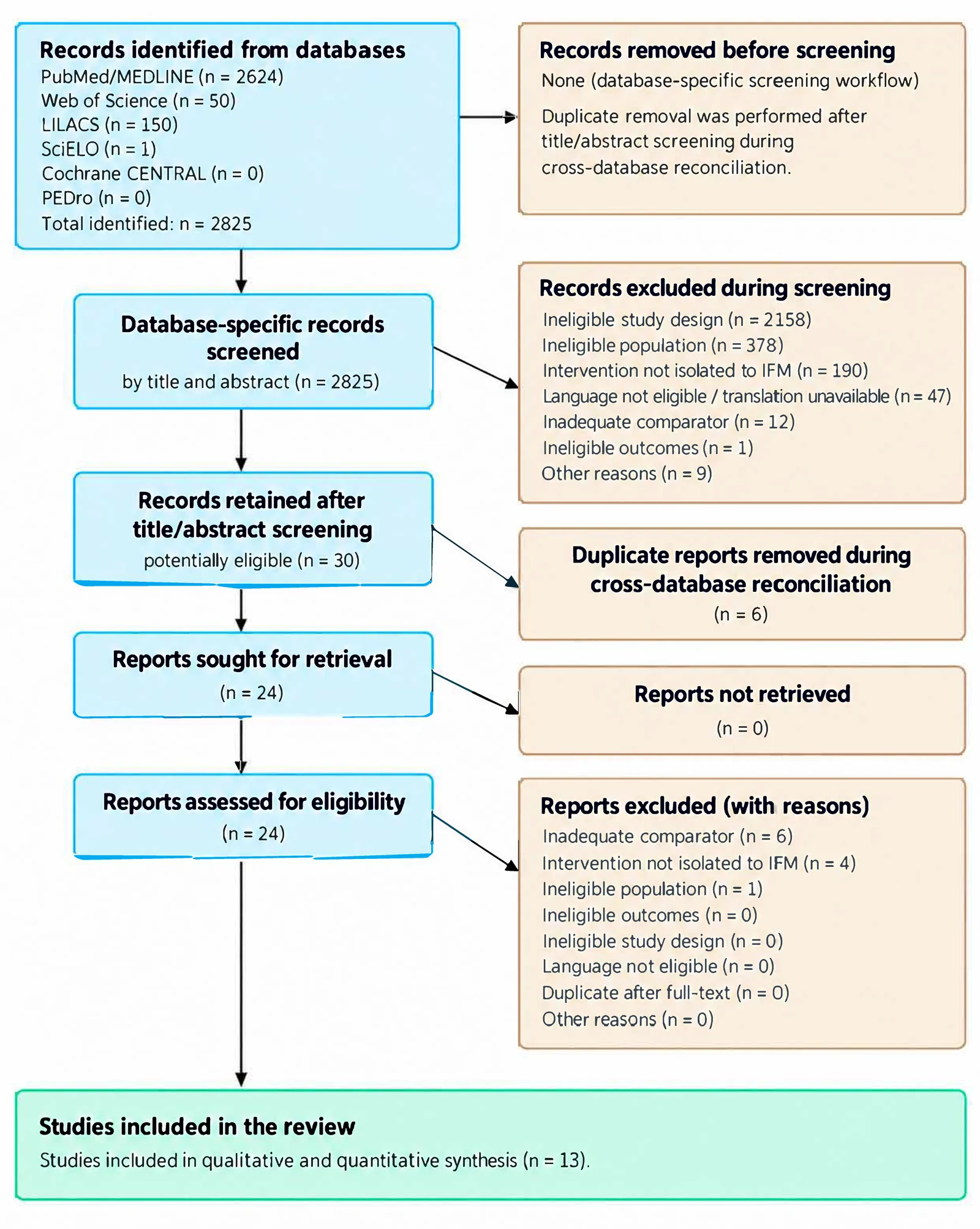
PRISMA 2020 flow diagram for the study selection process.

The final update searches executed on 5 June 2026 identified 2825 records across the six databases constituting the final reproducible search set: PubMed/MEDLINE (n = 2624), LILACS (n = 150), Web of Science (n = 50), SciELO (n = 1), Cochrane CENTRAL (n = 0), and PEDro (n = 0) (Figure 1). PubMed/MEDLINE accounted for 2624 of the 2825 records identified (92.9% of the total), whereas the searches conducted in Cochrane CENTRAL and PEDro returned no records under the database-specific strategies applied. These zero yields were retained as the actual database-specific search results and were not treated as missing searches or omitted sources. All 2825 records underwent database-specific title and abstract screening. Based on the archived screening reports, 2795 records were excluded at this stage for the following mutually exclusive primary reasons: ineligible study design (n = 2158), ineligible population (n = 378), intervention not isolated to the intrinsic foot muscles (n = 190), ineligible language or unavailable translation (n = 47), inadequate comparator (n = 12), ineligible outcomes (n = 1), and other reasons (n = 9). Thirty records remained potentially eligible after title/abstract screening. Cross-database reconciliation of these retained records identified six duplicate reports, leaving 24 unique reports for full-text retrieval and eligibility assessment.

Of the records excluded during title and abstract screening, 47 were classified as ineligible because they were published in languages other than English or Portuguese and/or a reliable translation was unavailable to the review team. No report reaching full-text assessment was subsequently excluded on the basis of language.

Twenty-four records were considered potentially eligible, and their full texts were retrieved. After full-text assessment, 11 studies were excluded for the following mutually exclusive reasons: intervention not isolated to the intrinsic foot muscles (n = 4), inadequate comparator (n = 6; studies in which the only available comparison involved another structured lower-limb exercise or strengthening, neuromuscular, or footwear-based intervention and no eligible non-strengthening control condition was available), and ineligible population (n = 1; participants aged outside the 18–59-year range). Multi-arm trials containing an eligible isolated IFM-training arm and an eligible control condition remained eligible even when additional active intervention arms were present; only the eligible IFM-versus-control contrast contributed to the synthesis of the isolated IFM effect. No study was excluded due to outcomes, study design, language, duplication, absence of extractable data, or other reasons.

Thirteen randomized controlled trials met all PICOS eligibility criteria: Day and Hahn [30], Fraser and Hertel [31], Gabriel et al. [32], Katsuya et al. [18], Lee and Choi [19], Lynn et al. [20], Okamura et al. [33], Ridge et al. [34], Suda et al. [35], Taddei et al. [36], Utsahachant et al. [37], Wei et al. [38], and Xiao et al. [39]. All studies meeting the final eligibility criteria were randomized controlled trials; no non-randomized, quasi-experimental, or grey-literature reports contributed to either the quantitative or narrative syntheses. The complete selection process is presented in the PRISMA 2020 flow diagram (Figure 1). Characteristics of included studies are described in Table 1; objectives, interventions, randomized between-group findings, and primary-study authors’ interpretations are presented in Table 2.

**Table 1:** Participant flow and characteristics across the included studies.

Author (Year)	Participant Flow (n)	Age (Years) Mean ± SD	Height Mean ± SD	Body Mass (kg) Mean ± SD	Training Status	Sex
Day and Hahn [30]	Randomized/analyzed: 20 Experimental: 11 CG: 9	Experimental: 24 ± 6 CG: 30 ± 12	Experimental 1.73 ± 0.10 m CG 1.72 ± 0.11 m	Experimental 60 ± 8 CG 62 ± 8	Competitive distance runners; >50 km/week; 5 km personal best <18:00 for men or <20:00 for women.	Both M/F counts: NR
Fraser and Hertel [31]	Reported total: 24 Analyzed: 23 Intervention: 11 CG: 12	Intervention: 23.6 ± 6.6 CG: 19.6 ± 1.2	Intervention 170.9 ± 11.5 cm CG 166.5 ± 13.8 cm	Intervention 70.5 ± 12.0 CG 64.9 ± 9.5	Healthy, recreationally active young adults; no history of foot or ankle injury; no previous experience with IFM exercises; ≥20 min/week of physical activity.	Both Intervention: 6 men, 5 women CG: 6 men, 6 women
Gabriel et al. [32]	Initially included: 30 Analyzed: 28 Active: 14 CG: 14	Active: 27.20 ± 3.49 CG: 24.50 ± 4.80	Active 177.00 ± 9.55 cm CG 175.00 ± 8.17 cm	Active 73.50 ± 14.10 CG 70.20 ± 13.80	Healthy, low-to-moderately active participants (≥5000–10,000 steps/day); did not use minimalist footwear; not professional athletes; no foot deformity.	Both Active 10 men 4 women CG 6 men 8 women
Katsuya et al. [18]	Randomized/analyzed: 24 Intervention: 12 CG: 12	Intervention: 19.4 ± 0.9 CG: 19.5 ± 1.1	Intervention 167.4 ± 8.6 cm CG 165.9 ± 8.0 cm	Intervention 61.4 ± 8.6 CG 61.7 ± 8.8	Patients with chronic ankle instability; physical activity level was not specifically reported.	Both Intervention 7 men 5 women CG 6 men 6 women
Lee and Choi [19]	Randomized/analyzed: 30 Experimental: 15 CG: 15	Experimental: 20.85 ± 1.09 CG: 20.80 ± 0.86	Experimental 166.42 ± 11.34 cm CG: 167.40 ± 10.60 cm	Experimental 62.14 ± 14.80 CG 67.00 ± 24.13	Adults with chronic ankle instability (CAI); ≥1 ankle sprain at least 1 year before enrollment; CAIT < 24; ≥2 episodes of ‘giving way’ in the preceding year.	Both Experimental 5 men 10 women CG 5 men 10 women
Lynn et al. [20]	Analyzed: 24 SFE: 8 TCE: 8 CG: 8	SFE: 23.7 ± 2.1 TCE: 22.8 ± 1.2 CG: 22.6 ± 1.7	SFE 1.72 ± 0.10 m TCE 1.69 ± 0.13 m CG 1.74 ± 0.10 m	SFE 69.9 ± 9.8 TCE 66.0 ± 11.2 CG 68.8 ± 9.4	Healthy university-aged volunteers; no history of major lower-limb pathology or balance impairment; activity level was not specified.	Both SFE 3 men 5 women TCE 4 men 4 women CG 3 men 5 women
Okamura et al. [33]	Randomized/analyzed: 20 Exercise: 10 CG: 10	Exercise: 20.2 ± 1.5 CG: 19.7 ± 0.9	Exercise 159.5 ± 8.8 cm CG 158.6 ± 6.1 cm	Exercise 53.7 ± 7.7 CG 49.7 ± 4.5	Individuals with pes planus (Foot Posture Index ≥ 6); physical activity level was not reported.	Both Exercise 1 man 9 women CG 2 men 8 women
Ridge et al. [34]	Recruited: 65 Analyzed: 57 FS: 19 MSW: 19 CG: 19	FS: 22 ± 1.8 MSW: 22.9 ± 3.3 CG: 22.6 ± 2.7	FS 175.2 ± 8.9 cm MSW 173.5 ± 9.3 cm CG 173.2 ± 10.9 cm	FS 69.4 ± 11.1 MSW 68.6 ± 10.9 CG 69.5 ± 13.3	Experienced runners aged 18–34 years; 15–30 miles/week for ≥6 months before the study; maintained mileage during the intervention.	Both FS 10 men 9 women MSW 9 men 10 women CG 11 men 8 women
Suda et al. [35]	Parent RCT randomized: 119 Analyzed in included report: 87 Intervention: 41 CG: 46	n: 40.3 ± 6.9 Intervention: 40.3 ± 7.7 CG: 40.3 ± 6.1	Intervention 166.5 ± 7.6 cm CG 171.8 ± 9.0 cm	Intervention 67.2 ± 12.1 CG 73.5 ± 13.0	Middle- and long-distance recreational runners; ≥1 year of running experience; mean training volume 35.8 ± 27.6 km/week; aged 18–55 years.	Both Intervention 17 men 24 women CG 25 men 21 women
Taddei et al. [36]	Randomized/analyzed: 28 Intervention: 14 CG: 14	Intervention: 41.9 ± 7.4 CG: 41.6 ± 6.0	Intervention 166.40 ± 7.80 cm CG 169.40 ± 9.18 cm	Intervention 68.3 ± 12.7 CG: 75.1 ± 13.9	Healthy recreational long-distance runners; aged 18–55 years; 20–100 km/week; not accustomed to minimalist footwear or barefoot running.	Both Intervention: 5 men, 9 women CG: 9 men, 5 women
Utsahachant et al. [37]	Randomized/analyzed: 45 SF: 15 SFLE: 15 CG: 15	SF: 21.00 (21, 29) SFLE: 21.00 (20, 22) CG: 21.00 (21, 26) Median (IQR)	SF 1.65 ± 0.07 m SFLE 1.66 ± 0.06 m CON 1.63 ± 0.06 m	SF 64.01 ± 8.82 SFLE 62.53 ± 13.34 CG 56.29 ± 12.22	Individuals with asymptomatic flexible flatfoot (navicular drop test > 10 mm); BMI ≤ 30 kg/m^2^; no lower-limb pain or injury in the previous 6 months; activity level was not reported.	Both SF: 5 men, 10 women SFLE: 5 men, 10 women CG: 5 men, 10 women
Wei et al. [38]	Randomized/analyzed: 40 Intervention: 20 CG: 20	Intervention: 21.0 ± 1.5 CG: 20.8 ± 1.8	Intervention 177.8 ± 6.0 cm CG 176.1 ± 5.4 cm	Intervention 72.2 ± 8.2 CG 71.1 ± 7.1	Professional non-rearfoot-strike runners; 10–15 km/week for ≥6 months before the study; no lower-limb injury in the previous 2 years; no experience with barefoot running or minimalist footwear.	Men
Xiao et al. [39]	Randomized/analyzed: 36 HD-tDCS: 12 FCE: 12 CG: 12	HD-tDCS: 21.9 ± 2.1 FCE: 22.7 ± 2.0 CG: 23.5 ± 1.5	HD-tDCS 174.9 ± 6.1 cm FCE 173.5 ± 7.1 cm CG 177.5 ± 6.1 cm	HD-tDCS 69.8 ± 7.6 FCE 69.3 ± 13.1 CG 75.2 ± 7.1	Young healthy male adults without a regular exercise habit; no history of lower-limb injury in the previous 6 months.	Men

Participant-flow values distinguish the initial sample from the analyzed sample when these differed in the source report. Sex distributions refer to the analyzed sample unless otherwise specified. For Suda et al. [35], the included publication was a secondary analysis of a larger parent RCT; 87 participants with complete biomechanical data contributed to the included report. NR: not reported; CAI: chronic ankle instability; CG: control group; FCE: foot core exercise; HD-tDCS: high-definition transcranial direct current stimulation; IFM: intrinsic foot muscles; IQR: interquartile range; SFE: short-foot exercise; SFLE: short-foot exercise plus lower-extremity training; TCE: towel-curl exercise.

**Table 2:** Characteristics, observed between-group findings, and primary-study authors’ interpretations of the included studies.

Study	Objective	Interventions	Observed Findings/ Authors’ Interpretation
Day and Hahn [30]	To investigate whether intrinsic foot muscle (IFM) strengthening alters running gait mechanics (metatarsophalangeal joint [MTPJ] and ankle kinetics/kinematics) and improves running economy (RE).	Experimental group (n = 11): 10-week IFM strengthening program including isometric, concentric and eccentric exercises (short-foot, foot curl, eccentric band curl and concentric band curl; 3 × 10 repetitions), performed once daily, three times per week, with weekly variation in ankle position (plantarflexion, neutral and dorsiflexion). Control group (n = 9): no additional strengthening protocol; participants maintained their usual running training. Assessments: baseline, 5 weeks and 10 weeks, including toe flexor strength, treadmill running mechanics on an instrumented treadmill, and RE assessed by indirect calorimetry.	**Observed between-group findings: **No extractable randomized between-group effect estimate with a 95% CI was available in the data extracted for toe-flexor strength. The experimental group increased toe-flexor strength by approximately 27% (*p* = 0.006), whereas the control group did not change (*p* = 0.607); these within-group changes were not treated as the randomized treatment effect. No intervention-related changes were reported for MTPJ/ankle mechanics or running economy. **Primary-study authors’ interpretation:** The authors concluded that gains in IFM strength did not translate into altered MTPJ/ankle mechanics or improved running economy in trained runners.
Fraser and Hertel [31]	To determine whether a progressive 4-week IFM exercise program improves motor function, assessed through clinical performance and perceived difficulty, and muscle activation measured using ultrasound imaging (USI) in healthy adults.	Intervention group (n = 11): progressive 4-week home-based program consisting of three exercises: toe-spread-out, hallux extension and lesser-toe extension; daily sessions with progressive loading using a TheraBand elastic band. Control group (n = 12): no additional intervention. Assessments: clinician-rated motor performance, perceived difficulty (Likert scale) and activation of the abductor hallucis (AbdH), flexor digitorum brevis (FDB) and flexor hallucis brevis (FHB) using USI during the three exercises.	**Observed between-group findings:** No significant group × time interaction was observed for AbdH activation during toe-spread-out, and no significant main effects or interactions were reported for the remaining comparisons. Toe-spread-out motor performance and perceived difficulty improved within the intervention group, but corresponding randomized between-group effect estimates and 95% CIs were not available in the extracted data and were not interpreted as standalone treatment effects. **Primary-study authors’ interpretation:** The authors interpreted the 4-week program as improving motor performance and reducing perceived exercise difficulty without clearly modifying ultrasound-derived muscle activation.
Gabriel et al. [32]	To investigate the local (foot), remote (posterior chain) and contralateral effects of a 4-week IFM exercise intervention in healthy recreationally active participants.	FE/active group (n = 14): five IFM-targeted exercises (short-foot, toe spread out, toe curl, hallux extension and lesser-toe extension), performed unilaterally (trained vs. untrained limb), 2 × 6 min, twice daily, at least 5 days/week for 4 weeks. Control group (n = 14): no intervention. Assessments: baseline (M1), post-intervention (M2) and after a 4-week washout (M3), including bilateral FPI-6, medial longitudinal arch mobility, single-leg balance, first MTP and ankle range of motion, posterior-chain flexibility, and performance.	**Observed between-group findings:** The extracted study summary primarily reported within-group changes in FPI-6, ankle range of motion, and balance; directly comparable randomized between-group effect estimates with 95% CIs were not available in the extracted data. FPI-6 decreased in the trained limb (−26%; *p* < 0.001) and untrained limb (−11%; *p* = 0.02) from M1 to M2, and the trained-limb change was maintained at M3. Posterior-chain outcomes, MTP range of motion, and ARI did not show significant change. These pre–post changes were not treated as equivalent to randomized between-group effects. **Primary-study authors’ interpretation:** The authors interpreted the intervention as producing predominantly local effects on foot posture, ankle range of motion, and balance, without clear remote effects on the posterior chain.
Katsuya et al. [18]	To examine the effects of a 4-week short-foot exercise (SFE) program on static and dynamic postural control and subjective ankle instability in individuals with chronic ankle instability (CAI).	Intervention group (n = 12): home-based SFE program, three times per week for 4 weeks; participants recorded each session on video (medial view of the foot) for technique review by the researchers. Control group (n = 12): maintained usual daily routines, with no additional training. Pre- and post-intervention assessments: static postural control (center-of-pressure excursion during single-leg stance), dynamic postural control (Star Excursion Balance Test [SEBT]) and subjective instability (Cumberland Ankle Instability Tool [CAIT]).	**Observed between-group findings:** Between groups, the SFE intervention produced greater improvement in static postural control (*p* < 0.05) and CAIT scores (*p* < 0.05) than the control condition. No significant between-group difference was observed for dynamic postural control measured with the SEBT (*p* > 0.05). Between-group effect estimates with 95% CIs were not available in the extracted data. **Primary-study authors’ interpretation:** The authors interpreted 4 weeks of SFE as improving static postural control and perceived ankle instability in individuals with CAI, but not dynamic postural control.
Lee and Choi [19]	To evaluate the effects of a 6-week IFM exercise program on muscle activation ratio, measured by ultrasound, and dynamic balance in adults with CAI.	Experimental group (n = 15): supervised IFM exercises, including toe spread out, first-toe extension and second-to-fifth-toe extension, three times per week for 6 weeks. Weekly progression: weeks 1–2 seated; weeks 3–4 bipedal standing; weeks 5–6 single-leg stance. Contraction durations were 3, 8 and 20 s; sets were 4 × 15, 4 × 8 and 4 × 3. Control group (n = 15): no exercise. Pre- and post-intervention assessments: activation ratio (contracted/resting thickness ratio using ultrasound) of the AbdH, FDB, FHB and quadratus plantae; dynamic balance assessed through composite reach distance, presumably using the SEBT.	**Observed between-group findings:** The randomized between-group comparison favored the experimental group for dynamic balance. Exact between-group effect estimates and 95% CIs were not available in the extracted data. Activation ratios increased within the experimental group for the assessed intrinsic foot muscles, but the available extraction did not provide corresponding between-group effect estimates for these ultrasound outcomes; the within-group changes were therefore not treated as standalone treatment effects. **Primary-study authors’ interpretation:** The authors interpreted the 6-week IFM program as improving intrinsic-foot-muscle activation and dynamic balance in participants with CAI.
Lynn et al. [20]	To investigate the effects of two different IFM training approaches (short-foot exercise vs. towel-curl exercise) on medial longitudinal arch height and performance during static and dynamic balance tasks in healthy young adults.	SFE group (n = 8 analyzed): short-foot exercise, 100 repetitions/day for 4 weeks (home-based). TCE group (n = 8 analyzed): towel-curl exercise, 100 repetitions/day for 4 weeks (home-based). Control group (n = 8 analyzed): no training. Pre- and post-intervention assessments: navicular height during weight bearing; mediolateral center-of-pressure (COP) excursion during a static-balance test; and mediolateral COP excursion during a separate dynamic-balance task.	**Observed between-group findings:** For the eligible SFE-versus-control contrast, no significant between-group differences were reported for navicular height or the static-balance task. During the dynamic-balance task, mediolateral COP movement on the dominant limb decreased by approximately 5 mm across all groups. The reported non-dominant-limb contrast favored SFE over TCE; because TCE was an additional active intervention arm, that contrast was not treated as the primary control comparison in this review. The quantitative synthesis used the eligible SFE-versus-control dynamic-COP data. **Primary-study authors’ interpretation:** The authors interpreted SFE as potentially more effective than TCE for dynamic-balance performance, with limb-specific differences. This interpretation was distinguished from the eligible SFE-versus-control contrast used in the present review.
Okamura et al. [33]	To evaluate the effect of plantar IFM strengthening exercises on static and dynamic foot kinematics, including dynamic navicular drop during gait, in individuals with pes planus.	Exercise group (n = 10): progressive short-foot exercise, three times per week for 8 weeks. The abstract did not provide detailed progression, although the intervention was described as progressive. Control group (n = 10): no intervention. Pre- and post-intervention assessments: static foot alignment (Foot Posture Index-6 and navicular drop test) and gait kinematics using 3D analysis, including dynamic navicular drop (difference between navicular height at heel contact and its minimum value) and time to reach the minimum value. The assessor was blinded to group allocation.	**Observed between-group findings:** The extracted study summary reported within-group improvement in FPI-6 calcaneal inversion/eversion scores (*p* < 0.05) and a reduction in the time to minimum navicular height during gait (*p* < 0.01). A directly extractable exercise-versus-control effect estimate with a 95% CI was not available in the extracted data; these within-group findings were therefore not interpreted as randomized treatment effects in isolation. **Primary-study authors’ interpretation:** The authors interpreted the findings as subtle improvements in static foot alignment and temporal gait-kinematic parameters and proposed improved windlass-mechanism function in individuals with pes planus.
Ridge et al. [34]	To determine the effect of minimalist-shoe walking versus isolated IFM strengthening exercises on foot muscle size and strength in runners.	FS/foot strengthening group (n = 19): progressive IFM strengthening exercises, including bilateral and unilateral heel raises on a flat surface and on a step edge, towel curls and toe spread; sets and repetitions progressed weekly across weeks 1–8; performed at least 5 days/week. MSW/minimalist-shoe walking group (n = 19): walking in minimalist footwear, with progressive increases in daily steps across 8 weeks. Control group (n = 19): maintained usual running mileage; no additional intervention. Assessments: size of seven foot/lower-leg muscles using ultrasound (ABDH, FDB, FDL, FHB, quadratus plantae, tibialis anterior and tibialis posterior) and strength using dynamometers (doming, great-toe flexion, and lesser-toe flexion) at weeks 0, 4 and 8.	**Observed between-group findings:** A significant overall group × time interaction was reported for all muscle-size and strength measures. The foot-strengthening group increased muscle size and strength from week 0 to week 8, whereas the control group showed no change; the additional minimalist-shoe-walking arm also improved. Exact foot-strengthening-versus-control effect estimates with 95% CIs were not consistently available in the extracted data. The minimalist-shoe arm was retained descriptively and was not treated as the primary comparator for the isolated IFM effect. **Primary-study authors’ interpretation:** The authors interpreted minimalist-shoe walking as being similarly effective to isolated strengthening for increasing foot muscle size and strength in runners; this comparison involved an additional active arm rather than the eligible control contrast used in this review.
Suda et al. [35]	To investigate the effects of a foot core muscle training program on interarticular foot coordination patterns (calcaneus-midfoot and midfoot-metatarsus) during running in recreational runners.	Intervention group (IG; n = 41): 8-week foot core training program (three times per week) comprising 12 exercises with progressive volume and difficulty. One weekly session was conducted with a physiotherapist, and three home sessions per week were remotely supervised using videos. Control group (CG; n = 46): placebo lower limb stretching protocol (5 min, three times per week), based on online descriptions and images. Assessments: barefoot running on an instrumented treadmill (9.5–11 km/h) with 3D kinematics using the Rizzoli foot model and 16 markers. Coordination between calcaneus-midfoot and midfoot-metatarsus coupling angles was analyzed in four joint angles using vector coding.	**Observed between-group findings:** Compared with the control group, the intervention group showed altered frontal-plane coordination during early stance (*p* = 0.020) and a higher frequency of transverse-plane anti-phase coordination with proximal dominance during propulsion (*p* = 0.013). An additional within-intervention change at heel contact was reported (*p* = 0.018). Effect estimates with 95% CIs were not available in the extracted data. These were biomechanical coordination outcomes; injury incidence was not measured. **Primary-study authors’ interpretation:** The primary-study authors proposed that the observed coordination changes might reduce running-related injury occurrence by increasing resistance to calcaneal pronation and producing a stiffer lever during push-off. This was an injury-prevention hypothesis, not an observed clinical effect, because injury incidence was not prospectively assessed.
Taddei et al. [36]	To investigate the effects of a foot strengthening program on IFM morphology and strength, and on running biomechanics, in healthy recreational long-distance runners.	Intervention group (IG; n = 14): 8-week program focused on IFM strengthening and activation during weight-bearing activities. Control group (CG; n = 14): no specific intervention. Assessments at baseline and 8 weeks: hallux and lesser-toe strength, foot function, cross-sectional area (CSA) and volume of the abductor hallucis (ABH), abductor digiti minimi (ABV), FDB and FHB using MRI; medial longitudinal arch range of motion and stiffness; and vertical and anteroposterior propulsive impulses during running.	**Observed between-group findings:** Compared with the control group, the intervention group showed increased volume in all investigated intrinsic foot muscles and increased vertical propulsive impulse during running. Correlations were reported between vertical propulsive impulse and ABH (r = 0.40), ABV (r = 0.41), and FDB volume (r = 0.69); these correlations were associations rather than randomized treatment effects. Exact between-group effect estimates with 95% CIs were not available in the extracted data. **Primary-study authors’ interpretation:** The authors interpreted the protocol as increasing IFM volume and propulsive forces and suggested a possible effect on running performance. This performance implication was an interpretation of the observed surrogate outcomes.
Utsahachant et al. [37]	To determine the effects of 6 weeks of short-foot exercise (SF), SF combined with lower-extremity training (SFLE), or a no-intervention control condition on dynamic foot function during gait in individuals with flexible flatfoot.	SF group (n = 15): short-foot exercise only, daily training for 6 weeks, delivered through telerehabilitation and home exercise; initial 30 min in-person instruction with a physiotherapist; supervision by videoconference three times per week. SFLE group (n = 15): short-foot exercise plus lower-limb muscle training (gluteal muscles, hip adductors, quadriceps, hamstrings and gastrocnemius); same 6-week structure. Control group (n = 15): no intervention. Pre- and post-intervention assessments: foot kinematics during gait, center-of-pressure excursion index (CPEI), intrinsic foot muscle test (clinical assessment of navicular control during single-leg stance) and navicular drop test (NDT).	**Observed between-group findings:** For the eligible isolated-SF-versus-control contrast, clinical IFM-test performance improved relative to control (SF vs. control: *p* < 0.001). Reductions in navicular drop and time to the lowest medial longitudinal arch were reported from baseline within the SF group, but corresponding between-group effect estimates with 95% CIs were not available in the extracted data. Greater CPEI change occurred in the SFLE arm versus SF and control; because SFLE included additional lower-extremity training, that active-arm contrast was retained descriptively and was not used to estimate the isolated IFM effect. **Primary-study authors’ interpretation:** The authors interpreted both exercise programs as improving dynamic foot function in flexible flatfoot, with greater CPEI change after SFLE than isolated SF. The SFLE comparison represents an additional active intervention rather than the primary isolated-SF-versus-control contrast of this review.
Wei et al. [38]	To investigate the effects of 8 weeks of IFM training on coordination patterns and coordination variability between the shank, rearfoot and forefoot during running in male runners with a non-rearfoot strike (NRFS) pattern.	Intervention group (n = 20): 8-week IFM strength training program, including foot stretching and massage plus 11 exercises (feet tapping, forefoot ascend, invert/evert asymmetric exercise, foot abduction, toe and ankle flexion, grab and hold a squeeze ball, squeeze toe separators, squeeze ball with little toes, toe abduction/adduction, short-foot and plantar arch raise). Sessions were conducted with a therapist and lasted approximately 40 min. Control group (n = 20): no intervention. Assessments: 3D running kinematics using the Rizzoli foot model with 16 reflective markers, at 12 km/h +/− 5%. A modified vector coding technique was used to analyze four joint-coupling pairs: FRTS, FRSF, FRFF and FRTF.	**Observed between-group findings:** Compared with the control group, IFM training produced greater responses for anti-phase coordination with proximal dominance in FRTS during early stance (*p* = 0.007; 95% CI: 2.75 to 15.43) and anti-phase coordination with distal dominance in FRSF during early stance (*p* = 0.025; 95% CI: 1.01 to 13.54). Additional coordination-pattern and coordination-variability changes were reported, although 95% CIs were not available for all reported comparisons. Injury incidence was not measured. **Primary-study authors’ interpretation:** The authors interpreted the changes as improved adaptive strategies and neuromuscular control and proposed potential implications for injury prevention. The injury-prevention statement is hypothesis-generating rather than an observed result because no prospective injury outcome was assessed.
Xiao et al. [39]	To examine the effects of 4 weeks of high-definition transcranial direct current stimulation (HD-tDCS) and foot core exercises (FCEs) on foot sensorimotor function (toe flexor strength and passive ankle kinesthesia) and postural control in healthy adult men.	HD-tDCS group (n = 12): 20 min of HD-tDCS (2 mA), three sessions per week for 4 weeks (12 sessions total). FCE group (n = 12): short-foot exercise, towel curls, toe spread and squeeze, and balance-board training, three sessions per week for 4 weeks (12 sessions total). Control group (n = 12): maintained usual activities, with no intervention. Pre- and post-intervention assessments: toe flexor strength, passive ankle kinesthesia (inversion/eversion) and postural control (center of gravity during single-leg stance with eyes open and closed).	**Observed between-group findings:** For the eligible FCE-versus-control contrast, MTP flexor strength (*p* = 0.027) and toe-flexor strength (*p* = 0.015) increased, and FCE produced greater reductions in mediolateral center-of-gravity sway velocity with eyes open (*p* = 0.033) and anteroposterior sway velocity with eyes closed (*p* < 0.001). Exact between-group effect estimates with 95% CIs were not available in the extracted data. HD-tDCS findings were retained as descriptive results from an additional active arm and were not used as the primary comparator for the isolated FCE effect. **Primary-study authors’ interpretation:** The authors interpreted 4 weeks of FCE as improving toe strength and postural control; they also described a different sensorimotor response profile for the additional HD-tDCS arm.

Note: For randomized trials, observed findings were summarized primarily from comparisons between the eligible IFM-targeted intervention and the eligible control condition. Within-group pre–post changes were retained only when needed to characterize the primary report and were not interpreted as evidence of a treatment effect in the absence of an appropriate between-group comparison. Between-group effect estimates and 95% confidence intervals are presented when available in the extracted primary-study data; unreported confidence intervals were not reconstructed. Additional active intervention arms in multi-arm trials are described for context but were not treated as control comparators for the isolated IFM effect. Statements labeled as primary-study authors’ interpretations represent the original investigators’ interpretation rather than observed effects. In particular, injury-prevention implications are hypothesis-generating because none of the included trials prospectively measured injury incidence. IFM: intrinsic foot muscles; SFE: short-foot exercise; CAI: chronic ankle instability; MTPJ: metatarsophalangeal joint; RE: running economy; COP: center of pressure; SEBT: Star Excursion Balance Test; CAIT: Cumberland Ankle Instability Tool; FPI-6: Foot Posture Index-6; MRI: magnetic resonance imaging; USI: ultrasound imaging.

The 13 included reports were published between 2012 and 2026 and comprised 462 participants in the analyzed samples, with analyzed sample sizes ranging from 20 to 87 participants per report. Initial enrollment or parent-trial randomization was larger in several reports because of attrition or because the included publication represented a secondary analysis of a larger RCT; these participant-flow differences are reported explicitly in Table 1. Eleven studies included participants of both sexes, whereas two included only men [38,39]. The investigated populations included recreational or competitive runners [30,34,35,36,38], individuals with chronic ankle instability [18,19], individuals with flatfoot or flexible flatfoot [33,37], and healthy recreationally active adults [20,31,32,39]. Intervention duration ranged from 4 to 10 weeks, with most protocols lasting between 4 and 8 weeks. Exercise type, weekly training frequency, and program duration were summarized descriptively across studies; because the outcome evidence was sparse and heterogeneous, these characteristics were not evaluated as formal effect moderators. These cross-study descriptions were not interpreted as moderator effects.

Although the eligibility criteria encompassed adults aged 18–59 years, the enrolled populations were predominantly young. In 11 of the 13 trials, group-level mean or median ages were approximately 19–30 years, whereas only two trials enrolled samples with mean ages around 40–42 years [35,36]. Accordingly, the available evidence does not provide balanced representation across the full 18–59-year adult age range and is dominated by younger and physically active or otherwise biomechanically selected populations. Sex-stratified intervention effects were not reported consistently across the included trials, and the available data did not permit a meaningful sex-specific quantitative or narrative synthesis. Accordingly, this review cannot determine whether responses to isolated IFM training differ between women and men.

### 3.2. Risk of Bias and Methodological Quality

**Figure 2:**
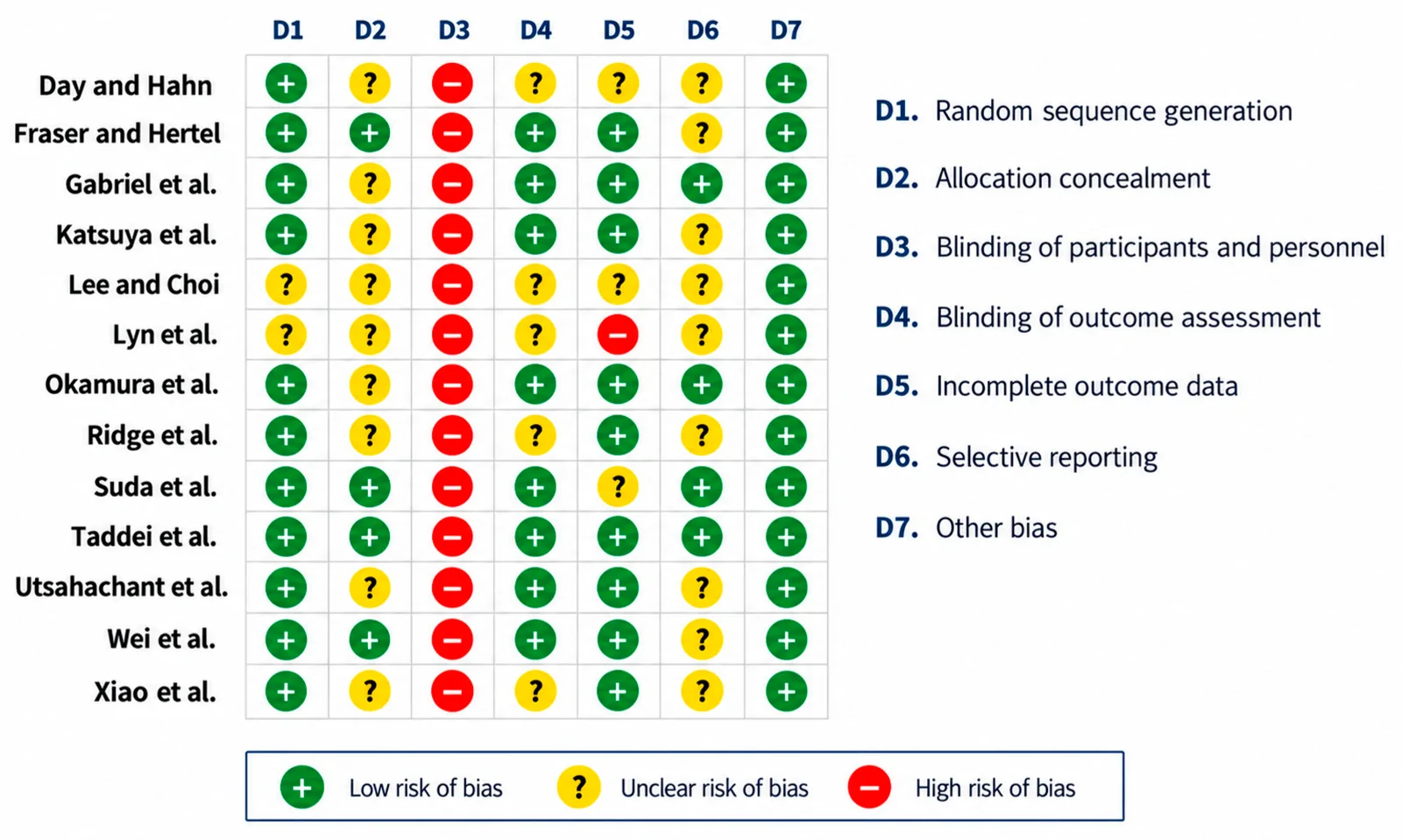
Domain-level descriptive risk-of-bias assessment of the included trials using the Cochrane RoB 1 tool. The display follows the seven standard RoB 1 domains. Exercise-specific considerations related to equipment, participant effort, and familiarization or learning were treated within the ‘other bias’ domain and not as independent Cochrane domains. No global study-level RoB 1 classification was assigned [18,19,20,30,31,32,33,34,35,36,37,38,39].

The descriptive RoB 1 assessment identified recurrent methodological concerns across the included trials (Figure 2). Random sequence generation was judged as low risk in 11 of 13 trials and unclear in two; allocation concealment was low risk in four trials and unclear in nine. Blinding of participants and personnel was judged as high risk in all 13 trials, reflecting the practical difficulty of blinding exercise-based interventions. Blinding of outcome assessment was low risk in eight trials and unclear in five. Incomplete outcome data were judged as low risk in nine trials, unclear in three, and high risk in one, whereas selective reporting was low risk in four trials and unclear in nine. The ‘other bias’ domain was judged as low risk across all 13 trials. These domain-level RoB 1 summaries were interpreted descriptively, and no global study-level RoB 1 classification was assigned; accordingly, the 13 trials should not be collectively characterized as being at uniformly high risk of bias.

**Figure 3:**
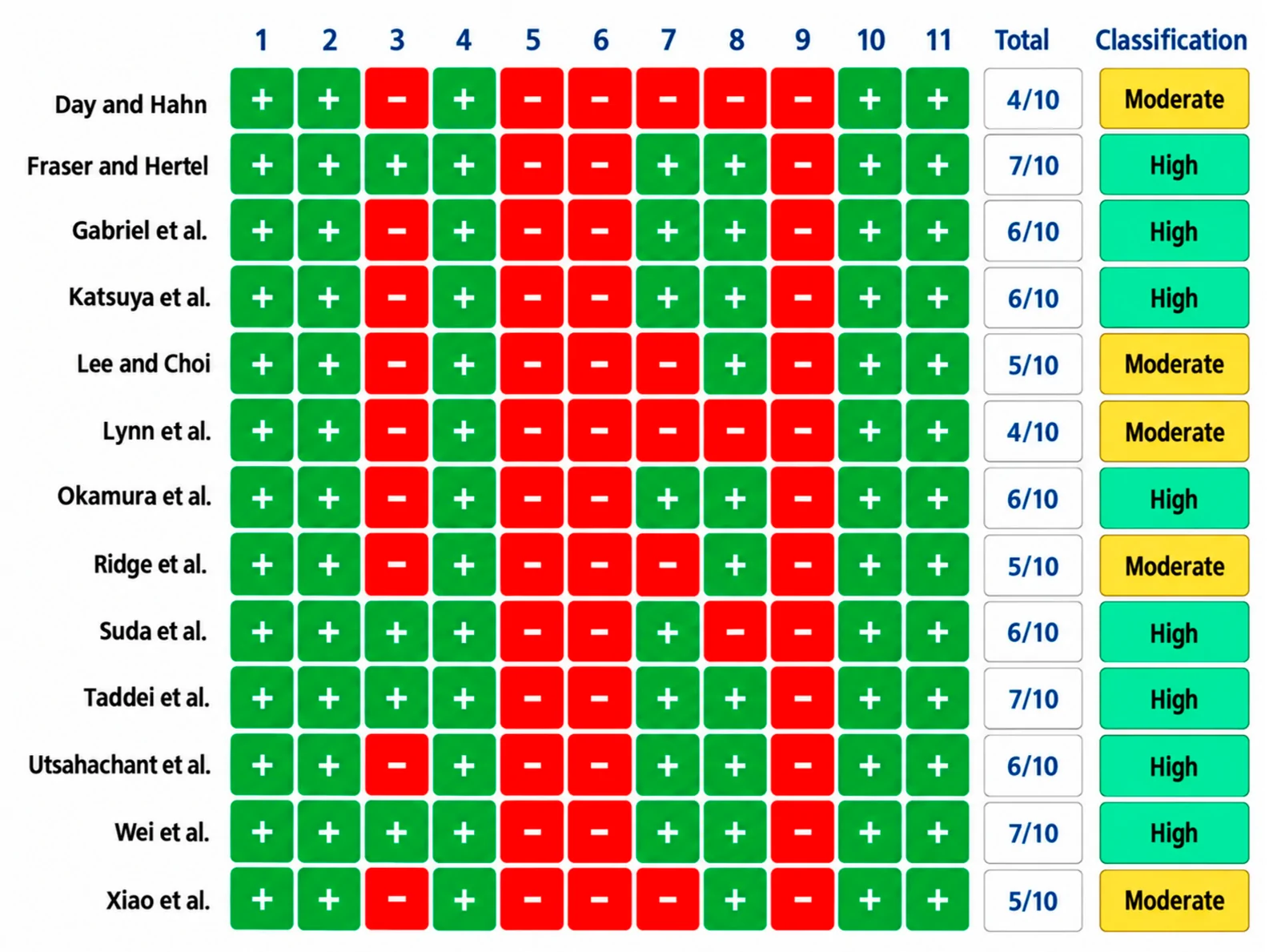
Methodological quality assessment of the included studies [18,19,20,30,31,32,33,34,35,36,37,38,39].

Methodological quality was additionally summarized using the PEDro scale. PEDro scale scores ranged from 4 to 7 points (median = 6; mean = 5.7 ± 1.0). Eight studies were classified as high methodological quality [18,31,32,33,35,36,37,38], and five as moderate methodological quality [19,20,30,34,39]. No study reached the excellent-quality category (≥9 points), and none was classified as poor quality (≤3 points). The item-level assessment is presented in Figure 3.

All 13 studies met criteria related to eligibility specification (item 1), random allocation (item 2), baseline comparability (item 4), between-group statistical comparisons (item 10), and reporting of point estimates and variability measures (item 11).

The main methodological limitations were concentrated in four domains. First, only four studies (30.8%) explicitly described allocation concealment procedures (item 3): Fraser and Hertel [31], Suda et al. [35], Taddei et al. [36], and Wei et al. [38] reported the use of sealed opaque envelopes or allocation conducted by an independent researcher. Second, none of the studies (0%) blinded participants (item 5) or therapists delivering the intervention (item 6), an inherent feature of exercise-based trials [21,40]. Assessor blinding (item 7) was reported in eight studies (61.5%) [18,31,32,33,35,36,37,38]. Third, none of the studies (0%) conducted an intention-to-treat analysis (item 9). Fourth, Suda et al. [35] refers to a secondary analysis including 87 of 119 originally randomized participants (73.1%), below the 85% retention threshold required for item 8 of the PEDro scale.

In summary, PEDro scores varied across the included trials, but important internal-validity concerns remained across the evidence base. These concerns included incomplete reporting of allocation concealment, lack of participant and therapist blinding, variable assessor blinding, and absence of explicit intention-to-treat analyses. Because PEDro and Cochrane risk-of-bias tools assess overlapping but conceptually distinct aspects of trial methodology, PEDro scores were not used to override or attenuate the outcome-specific risk-of-bias judgments. Relatively higher PEDro scores should therefore not be interpreted as evidence that a trial was necessarily at low risk of bias.

For dynamic postural control, the only secondary outcome eligible for quantitative synthesis, risk of bias for the pooled evidence was evaluated at the result level using RoB 2 rather than inferred from the descriptive study-level RoB 1 domain summaries. This distinction was particularly important because lack of participant and therapist blinding, although common and often unavoidable in exercise trials, does not necessarily have the same implications for all outcomes. For dynamic postural control, judgments therefore considered whether awareness of intervention assignment resulted in deviations from the intended interventions, whether outcome assessors were aware of allocation and whether the performance-based measurements were susceptible to such awareness, the completeness of outcome data, and the possibility of selective reporting. Overall, the RoB 2 evaluation identified some concerns across all three contributing trials, primarily related to incomplete reporting of randomization or allocation procedures, aspects of outcome assessment, and the ability to verify selection of the reported result against prespecified analyses. Missing outcome data were limited or absent for the results contributing to the meta-analysis. The domain-level judgments and overall RoB 2 classifications are summarized together in Table 3. These outcome-specific judgments, rather than the descriptive study-level RoB 1 domain summaries, were considered when evaluating the risk-of-bias domain within GRADE for the pooled dynamic postural-control outcome.

### 3.3. Primary Clinical Outcomes: Ankle Injury, Ankle-Instability Events, and Knee-Related Clinical Endpoints

None of the 13 included randomized controlled trials reported any predefined primary clinical endpoint. No trial prospectively reported incident or recurrent lateral ankle sprains. Likewise, no trial prospectively recorded giving-way episodes as incident ankle-instability events. These ankle endpoint categories were treated separately because a giving-way episode may reflect functional instability without constituting a new traumatic injury. Within the knee domain, no trial reported incident acute knee injuries, and no trial evaluated the new onset or incidence of knee-related symptoms or clinical syndromes such as patellofemoral pain syndrome, patellar tendinopathy, or iliotibial band-related symptoms. Accordingly, both ankle endpoint categories and both conceptually distinct knee endpoint categories resulted in empty syntheses. Neither quantitative pooling nor comparative narrative synthesis was possible. Consequently, no direct estimate of the effect of isolated IFM training on these primary clinical outcomes could be generated from the available randomized evidence.

### 3.4. Secondary Surrogate Outcomes

Because the predefined primary-outcome synthesis was empty, the remaining analyses addressed secondary functional, biomechanical, and neuromuscular surrogate outcomes.

#### 3.4.1. Dynamic Postural-Control Domain: Meta-Analysis

Risk-of-bias findings for this pooled outcome are reported in Section 3.2 and Table 3; all three contributing trials were judged as having some concerns overall in the result-specific RoB 2 assessment.

The primary quantitative synthesis combined three studies that operationalized the broader domain of dynamic postural control using related, but not interchangeable, measures. Katsuya et al. [18] and Lee and Choi [19] quantified dynamic balance using reach-based outcomes in participants with chronic ankle instability, whereas Lynn et al. [20] quantified mediolateral COP excursion during a dynamic-balance task in healthy university-aged adults. The separate static-balance COP result reported by Lynn et al. was not included in the meta-analysis. These measures were retained within the same broader domain because each assessed postural control during a dynamic balance challenge, but they were not considered metrically or constructively equivalent. Outcome directions were harmonized before calculation of standardized mean differences as described above. Baseline dynamic postural-control values were reviewed in the contributing trials where they were reported. The available reports did not indicate a consistent pattern of baseline imbalance favoring either intervention or control groups; however, reporting was not sufficiently complete or homogeneous to calculate baseline-adjusted standardized effects across all three studies. In particular, Lee and Choi [19] reported similar pre-intervention composite reach values between the experimental and control groups (66.79 ± 9.64% and 65.44 ± 8.67% of leg length, respectively).

**Figure 4:**
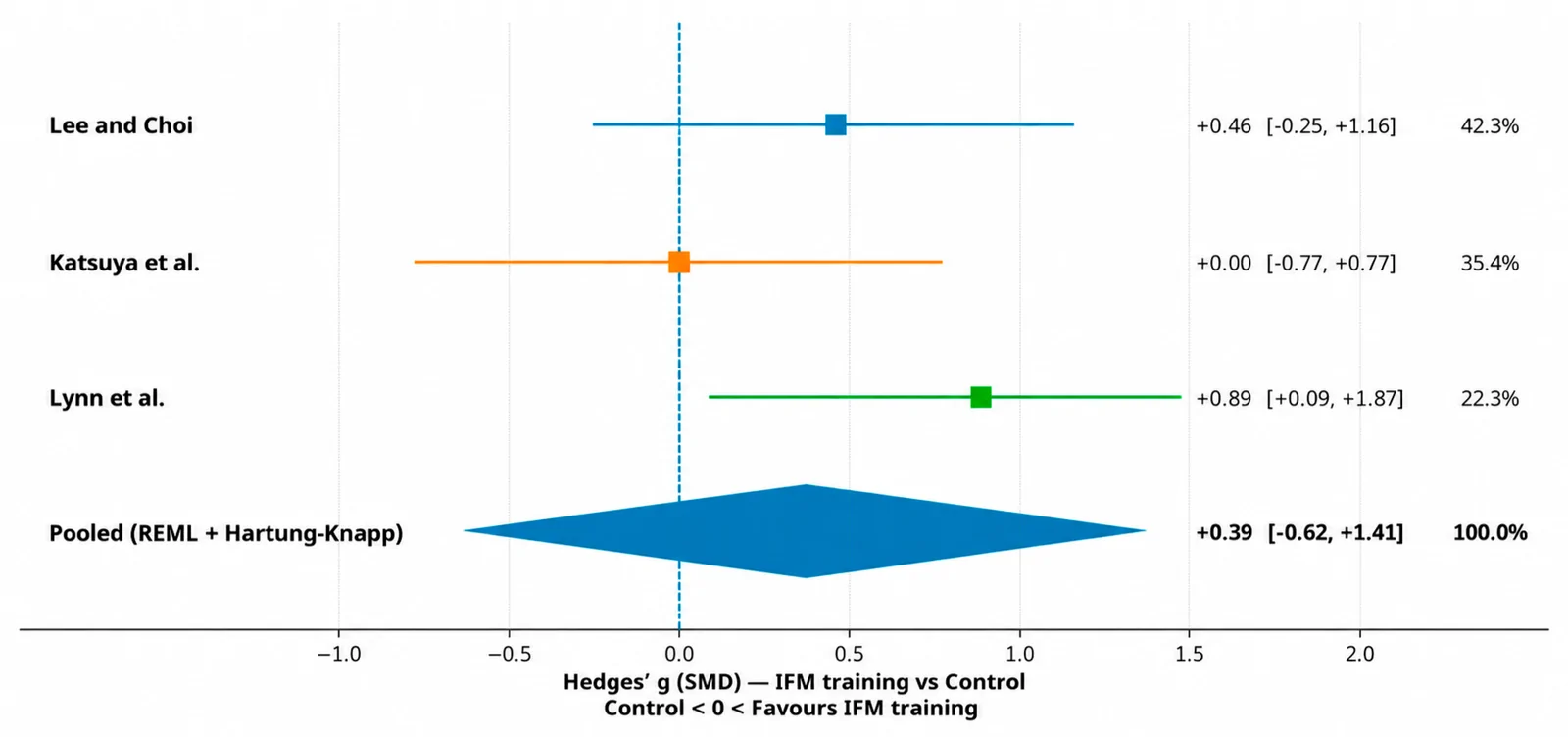
Forest plot of the effects of isolated intrinsic foot muscle (IFM) training on dynamic postural control. Individual and pooled effects are expressed as Hedges’ g with 95% confidence intervals. The pooled estimate was obtained using a random-effects model with restricted maximum likelihood (REML) estimation of between-study variance and Hartung–Knapp adjustment for the confidence interval. Positive values favor IFM training. The 95% prediction interval for the random-effects model is reported in the accompanying Results section and is interpreted cautiously because k = 3 [18,19,20].

Because corresponding change-score standard deviations or equivalent derivation data were not consistently available across all three studies, a pooled change-score sensitivity analysis could not be conducted without imputing unknown pre–post correlations. Such imputation was deliberately avoided. Using a random-effects model with REML estimation and Hartung–Knapp adjustment, the pooled effect estimate favored IFM training for dynamic postural control (Hedges’ g = 0.39), but the estimate was highly uncertain and the 95% confidence interval (−0.62 to 1.41) was compatible with effects ranging from potentially unfavorable or negligible to potentially meaningful benefit (*p* = 0.238; Figure 4). Individual study estimates ranged from g = 0.00 [18] to g = 0.89 [20]. The observed statistical heterogeneity was I^2^ = 1.3% (Q = 2.03; df = 2; *p* = 0.36; τ^2^ ≈ 0.002). The corresponding 95% prediction interval was approximately −2.66 to 3.44. This very wide interval spans potentially unfavorable, negligible, and large beneficial effects and should be regarded as an exploratory estimate rather than a stable characterization of the distribution of true effects, because it is based on only three studies and an imprecisely estimated between-study variance. Although this value is numerically low, it should be interpreted with substantial caution because only three small studies contributed to the meta-analysis. With such a limited number of studies, both Cochran’s Q and I^2^ have low power and limited precision for detecting true between-study heterogeneity. Therefore, the low observed I^2^ was not considered evidence of clinical, methodological, or construct homogeneity. The wider Hartung–Knapp confidence interval encompassed both negligible and potentially meaningful effects and therefore reinforced the imprecision of the pooled estimate. A sensitivity analysis using the DerSimonian–Laird estimator yielded a similar point estimate (Hedges’ g = 0.39) with a narrower 95% CI (−0.07 to 0.86) and did not alter the direction or statistical interpretation of the pooled effect. In the post hoc population-and-measurement sensitivity analysis excluding Lynn et al. [20], only the two chronic ankle instability trials using reach-based dynamic-balance outcomes remained [18,19]. This analysis simultaneously removed the healthy cohort and the only trial contributing a dynamic COP-excursion measure rather than a reach-distance measure. Given k = 2, a separate random-effects pooled estimate was not emphasized; descriptively, the remaining trials did not provide a consistent signal that materially altered the interpretation of the primary synthesis.

The certainty of evidence for dynamic postural control was rated as Very Low according to GRADE (Table 4). Randomized evidence started at High certainty and was downgraded one level for risk of bias because all three trials were judged as having some concerns in the outcome-specific RoB 2 assessment, one level for indirectness because of differences in populations and outcome operationalization and because dynamic postural control is a surrogate rather than an injury-incidence outcome, and one level for serious imprecision because only three small trials (n = 70) contributed to the analysis and the Hartung–Knapp confidence interval was wide (Hedges’ g = 0.39; 95% CI −0.62 to 1.41). Inconsistency was not downgraded; although I^2^ was numerically low, this estimate was interpreted cautiously given the small number of studies. Publication bias could not be formally assessed with k = 3 and was not downgraded because there was insufficient evidence to support an additional rating reduction. Consequently, the evidence is very uncertain regarding the effect of isolated IFM training on dynamic postural control. The five GRADE domain judgments are reported formally in Table 4.

**Table 4:** Summary of findings and GRADE certainty assessment.

Outcome	Studies/Participants	Effect	Risk of Bias	Inconsistency	Indirectness	Imprecision	Publication Bias	Overall Certainty
Dynamic postural control	3 RCTs/70	Hedges’ g = 0.39 95% CI −0.62 to 1.41	Serious (−1)	Not serious	Serious (−1)	Serious (−1)	Not downgraded	Very Low
Incident ankle injury	0 studies	Not estimable	Not rated	Not rated	Not rated	Not rated	Not rated	Not rated
Incident ankle-instability events (giving way)	0 studies	Not estimable	Not rated	Not rated	Not rated	Not rated	Not rated	Not rated
Incident acute knee injury	0 studies	Not estimable	Not rated	Not rated	Not rated	Not rated	Not rated	Not rated
Incident knee-related symptoms/clinical syndromes	0 studies	Not estimable	Not rated	Not rated	Not rated	Not rated	Not rated	Not rated

RCTs: randomized controlled trials; CI: confidence interval.

GRADE certainty ratings: High, Moderate, Low, and Very Low. Randomized evidence starts at High certainty. Dynamic postural control was downgraded one level for risk of bias (all three contributing trials had some concerns in the outcome-specific RoB 2 assessment), one level for indirectness (clinical population differences, related but non-interchangeable outcome measures, and the surrogate nature of dynamic postural control), and one level for serious imprecision (k = 3; n = 70; wide Hartung–Knapp 95% CI spanning negligible, potentially unfavorable, and potentially meaningful beneficial effects). Inconsistency was not downgraded. Publication bias was not formally assessable with three studies and was not downgraded because there was insufficient evidence to justify an additional reduction. The four predefined primary clinical outcome categories were not rated because no eligible randomized trial reported these endpoints, and no body of evidence was available for formal GRADE assessment.

#### 3.4.2. Other Functional, Neuromuscular, and Biomechanical Outcomes: Narrative Synthesis

The secondary outcome domains that did not meet the criteria for quantitative pooling were synthesized using a structured SWiM approach. For each outcome cluster, we considered the number of contributing studies and participants, the direction and approximate magnitude of effects, consistency across studies, relevant risk-of-bias concerns, and the principal limitations affecting interpretation. Statistical significance was not used as the primary basis for determining whether an intervention effect was present. Instead, emphasis was placed on the direction, magnitude, and consistency of observed effects and on the methodological context of the contributing evidence (Table 5).

Participant counts refer to the eligible IFM-versus-control comparison(s) contributing to each outcome cluster rather than the total randomized sample in multi-arm studies. For narratively synthesized outcomes, the RoB 1 assessment was used only to describe recurrent study-level methodological limitations and was not interpreted as a formal result-specific risk-of-bias judgment. Formal RoB 2 assessments were not undertaken for these sparse and heterogeneous narrative outcome clusters; their methodological limitations are therefore described qualitatively. No formal GRADE certainty rating was assigned to these sparse narrative outcome clusters; limitations are therefore described qualitatively rather than as GRADE categories.

Across local foot-muscle and structural outcomes, the direction of evidence generally favored IFM-targeted training, although the magnitude and comparability of the reported effects varied. Foot muscle strength was represented by four RCTs (110 participants in eligible IFM-versus-control comparisons) [30,34,36,39]. Selected strength measures improved by approximately 13–37%; however, comparable between-group effect estimates were not consistently extractable, and local strength gains did not consistently translate into higher-level biomechanical or performance changes. Foot muscle morphology was examined in two RCTs (66 participants) [34,36] and showed a favorable direction, with increases in intrinsic foot muscle size or volume assessed using ultrasound or MRI. Cross-study comparison remained limited by differences in imaging methods and the specific muscles evaluated. Foot posture was assessed in three RCTs (78 participants) [32,33,37]. Findings generally favored IFM training, but the absolute changes were small—commonly approximately 0.5–1.5 FPI points or 1–2 mm of navicular drop—and some changes approached measurement error or minimal detectable change, limiting clinical interpretation.

For postural and sensorimotor outcomes that were not meta-analyzed, static postural control was assessed in three RCTs (76 participants) [18,32,39]. Findings generally favored IFM training for selected single-leg balance and center-of-pressure/center-of-gravity measures, but the tasks and metrics differed across studies, limiting direct comparability. Subjective ankle instability was represented by one RCT (24 participants) [18] and favored IFM training according to CAIT scores; however, consistency could not be assessed and the self-reported nature of the outcome increases susceptibility to expectation effects in an unblinded exercise trial. Ankle kinesthesia/proprioception was also represented by a single eligible IFM-versus-control comparison (24 participants) [39], with favorable changes in selected sensorimotor measures. Because this evidence came from a single small study, reproducibility and external validity remain uncertain.

Higher-level biomechanical and motor-performance outcomes were less consistent. Inter-articular running coordination was evaluated in two RCTs (127 participants) [35,38]. Both studies reported changes in coupling/coordination patterns or reduced coordination variability compatible with a favorable mechanistic direction, but the vector-coding variables differed and neither trial prospectively assessed injury incidence. Running mechanics and running economy were evaluated in two RCTs (48 participants) [30,36] and yielded mixed findings: Taddei et al. [36] reported an increase in vertical propulsive impulse, whereas Day and Hahn [30] found no meaningful change in joint mechanics or running economy despite increases in toe-flexor strength. Motor performance/perceived exercise difficulty was represented by one RCT (23 analyzed participants) [31]; toe-spread-out performance and perceived difficulty improved, whereas ultrasound-derived muscle activation did not show a clear between-group improvement. Overall, the non-pooled evidence suggests possible local and task-specific adaptations, but small evidence clusters, heterogeneous measurement approaches, qualitative risk-of-bias concerns, and the absence of direct clinical endpoints preclude conclusions about reproducible clinical effectiveness or injury-preventive benefit.

## 4. Discussion

The principal finding of this systematic review is that the evidence base is empty for its predefined primary clinical outcomes. None of the 13 randomized controlled trial reports, comprising 462 participants in the analyzed samples, reported incident ankle injuries, prospectively recorded ankle-instability events, incident acute knee injuries, or incident knee-related symptoms/clinical syndromes. Therefore, no direct evidence is currently available to determine whether isolated IFM training affects these distinct primary clinical endpoints in adults. This absence of primary-outcome evidence is distinct from evidence of no preventive effect: rather, the relevant clinical outcomes have not been tested in the available randomized literature. The evidence that could be synthesized concerned only secondary surrogate outcomes. Dynamic postural control was the sole secondary outcome cluster eligible for meta-analysis. The pooled estimate favored IFM training in direction (Hedges’ g = 0.39), but the effect was highly uncertain, with a wide Hartung–Knapp 95% confidence interval (−0.62 to 1.41) spanning potentially unfavorable, negligible, and potentially meaningful beneficial effects (*p* = 0.238). The certainty of this evidence was rated Very Low because of concerns regarding risk of bias, indirectness, and serious imprecision. Accordingly, the pooled estimate should not be interpreted as reliable evidence either for or against an effect of isolated IFM training on dynamic postural control. Other individual trials reported changes in foot muscle strength, morphology, foot posture, proprioception, subjective ankle instability, and inter-articular coordination. Although these adaptations may be mechanistically relevant to injury prevention, they cannot be assumed to translate into fewer ankle or knee injuries.

### Absence of Direct Injury-Prevention Evidence

The absence of trials measuring injury incidence represents more than a limitation of statistical pooling; it identifies a fundamental disconnect between the proposed preventive rationale for IFM training and the outcomes currently used to evaluate it. Existing trials have predominantly focused on mechanistic or intermediate endpoints measured over 4–10 weeks, whereas injury prevention requires prospective surveillance over substantially longer periods. Improvements in balance, muscle strength, foot morphology, or intersegmental coordination are biologically plausible mediators of reduced injury risk, but they remain surrogate outcomes unless their relationship with subsequent injury incidence is empirically demonstrated. Within the ankle domain, giving-way episodes were distinguished from incident sprains because they represent episodes of functional instability and may occur in the absence of a new traumatic injury. Accordingly, self-reported ankle-instability scores, such as CAIT scores, were treated as secondary surrogate outcomes and were not considered equivalent to prospectively observed giving-way events.

The biological rationale for IFM training as a preventive strategy is theoretically grounded in the foot core paradigm proposed by McKeon et al. [5], although its clinical translation remains empirically unconfirmed. According to this model, the IFM, as local stabilizers of the medial longitudinal arch, modulate subtalar pronation, contribute to midfoot stiffness during late stance, and provide afferent input from plantar mechanoreceptors essential for postural control. Mechanistic studies have shown progressive IFM recruitment as locomotor demand increases [41], and the IFM contribute to elastic energy storage and return during running [42,43]. In this review, Taddei et al. [36] reported increases in the volume of the measured IFM together with an increase in vertical propulsive impulse, whereas Suda et al. [35], in a separate sample, reported modifications in inter-articular coordination patterns at the calcaneus–midfoot and midfoot–metatarsal couplings. Considered together, these findings are compatible with a possible mechanistic hypothesis in which IFM-targeted training may influence both local muscular characteristics and select aspects of lower-limb biomechanical function. However, because these observations were obtained from different studies and independent samples, they do not demonstrate that morphological adaptations caused or mediated the observed changes in inter-articular coordination or biomechanical control.

The quantitative synthesis should be interpreted cautiously because the three pooled studies did not operationalize dynamic postural control identically. Katsuya et al. [18] and Lee and Choi [19] contributed reach-based dynamic-balance outcomes, whereas Lynn et al. [20] contributed mediolateral COP excursion recorded during a dynamic-balance task; Lynn et al.’s separate static-balance COP measure was not pooled. These outcomes capture related aspects of postural regulation, but they are not interchangeable metrics. They were grouped because each was considered to represent performance within the broader latent domain of dynamic postural control during a challenging balance task; nevertheless, standardized mean differences address differences in scale and direction, not differences in construct validity. Accordingly, the pooled estimate should be interpreted as a broad summary of dynamic postural-control performance rather than as the effect of IFM training on a single standardized balance test. Accordingly, the decision to pool these studies should not be interpreted as evidence that their measurement approaches were equivalent. Rather, pooling represented a pragmatic domain-level synthesis after clinical, methodological, and construct comparability had been judged sufficient, while the remaining measurement and population differences were incorporated into the interpretation of the pooled estimate and the GRADE indirectness judgment.

Clinical heterogeneity was also substantial: Lee and Choi [19] and Katsuya et al. [18] recruited individuals with chronic ankle instability, whereas Lynn et al. [20] recruited healthy university-aged adults, populations that differ in baseline sensorimotor impairment and potential for improvement. The primary meta-analysis used post-intervention values to preserve the randomized between-group contrast and avoid introducing assumptions about unreported pre–post correlations. Baseline outcome values were reviewed where available, and no consistent pattern of baseline imbalance was evident; however, reporting across the three trials was insufficient to derive comparable baseline-adjusted standardized estimates or to perform a robust pooled change-score sensitivity analysis without imputing unknown correlations. This limitation should be considered when interpreting the pooled estimate. The observed I^2^ of 1.3% should therefore not be interpreted as evidence that the studies were homogeneous. With only three small trials, heterogeneity statistics have limited power and precision, and the absence of a large I^2^ may reflect insufficient information to detect between-study variability rather than true homogeneity. Clinical and construct heterogeneity should consequently be judged independently of the numerical I^2^ estimate. Consistent with this uncertainty, the 95% prediction interval was extremely wide (approximately −2.66 to 3.44), indicating that the effect observed in a future comparable trial could plausibly vary substantially in either direction. However, with k = 3, the prediction interval is itself highly unstable because both the pooled mean effect and between-study variance are estimated from very limited information; it should therefore be interpreted as a descriptive illustration of uncertainty rather than a precise forecast. A post hoc sensitivity analysis excluding the healthy cohort left only the two chronic ankle instability trials; this restricted analysis was interpreted descriptively because k = 2 and did not provide a sufficiently robust basis for a separate confirmatory pooled estimate. Importantly, reanalysis using REML with Hartung–Knapp adjustment produced a pooled estimate of similar magnitude and direction to the original DerSimonian–Laird model but substantially wider confidence limits. This greater uncertainty reinforces that the available quantitative evidence is too imprecise to establish either a clinically meaningful benefit or equivalence. The wider confidence interval also supported downgrading for imprecision in the GRADE assessment.

The narrative synthesis of remaining outcome clusters must be interpreted with caution, as quantitative pooling was not feasible. For foot muscle strength, the only study providing extractable data for between-group comparison demonstrated a significant 13% gain in toe flexor strength [39], whereas Taddei et al. [36] reported a 37% increase that did not reach statistical significance in the group-by-time interaction. Critically, Day and Hahn [30] demonstrated that strength gains over 10 weeks did not transfer to changes in joint mechanics or running economy, a dissociation consistent with the task-specificity of strength adaptations [44]. For foot posture and morphology, four trials reported improvements in the Foot Posture Index, navicular drop, and muscle cross-sectional area following 4 to 10 weeks of training; however, the magnitude of postural changes, typically 0.5–1.5 FPI points or 1–2 mm of navicular drop, approaches the minimal detectable change for these instruments, limiting clinical interpretability. Accordingly, statistically detectable changes in these postural measures should not automatically be interpreted as clinically meaningful improvement. When the magnitude of change approaches the measurement error or minimal detectable change in the instrument, the practical relevance of the observed effect remains uncertain even when conventional statistical significance is reached. Notably, Gabriel et al. [32] demonstrated that adaptations were local and did not propagate to the posterior chain, constraining the rationale for prescribing isolated IFM training as a global lower-limb prehabilitation strategy. For inter-articular coordination, Suda et al. [35] and Wei et al. [38] used vector coding to demonstrate improved rearfoot–midfoot coupling and reduced coordination variability after 8 weeks, the most mechanistically direct findings in this review, although neither study assessed injury incidence, and the inferential link between improved coordination and reduced injury risk remains empirically untested [45].

The structured narrative synthesis of the remaining outcome clusters should be interpreted cautiously because the evidence was sparse, heterogeneous, and generally based on one or two studies per domain. Across studies of foot muscle strength, the direction of effect generally favored IFM training, with reported improvements of approximately 13% to 37% in selected strength measures; however, comparable between-group effect estimates were not consistently available, and local strength gains did not uniformly translate into higher-level biomechanical or performance changes. Foot posture and morphology also showed predominantly favorable directions, but postural changes were small, commonly about 0.5–1.5 FPI points or 1–2 mm of navicular drop, and may approach measurement error or minimal detectable change for some instruments. Inter-articular coordination findings from Suda et al. [35] and Wei et al. [38] were directionally consistent with altered rearfoot–midfoot or shank–foot coordination and reduced variability, but the specific vector-coding variables differed and neither study measured subsequent injury incidence. Running-level outcomes were less consistent: Taddei et al. [36] reported increased vertical propulsive impulse, whereas Day and Hahn [30] found no meaningful change in joint mechanics or running economy despite increased toe-flexor strength. Taken together, these findings suggest possible task-specific and mechanistic adaptations, but the magnitude and clinical relevance of the non-pooled effects remain uncertain. This distinction between statistical detectability and clinical meaningfulness is particularly important for surrogate biomechanical outcomes, for which small numerical changes may not exceed measurement variability or translate into perceptible functional or injury-related benefit.

These findings should be situated within the broader context of prior syntheses. Jaffri et al. [12] and Wei et al. [11] reported moderate-to-large effects on navicular drop, dynamic balance, and plantar strength, while Peters-Dickie et al. [13] extended these findings to minimalist footwear with low-to-very-low GRADE certainty. Rather than representing the first synthesis of IFM-targeted exercise effects, the present review extends previous evidence by narrowing the intervention contrast, explicitly prioritizing ankle injury incidence and conceptually distinct knee-related clinical endpoints as predefined outcomes, and separating these clinical endpoints from surrogate biomechanical, neuromuscular, and functional outcomes. It also applied an additional conservative operational threshold of k ≥ 3 only after clinical, methodological, and construct comparability had been established; this threshold was specific to the present review and was not treated as a sufficient criterion for pooling. These methodological choices were intended to increase the specificity and interpretability of the review question and to reduce one source of intervention heterogeneity; however, they should not be interpreted as inherently increasing internal validity, which remains dependent on the design and conduct of the included trials and on the extent to which the IFM-specific contribution can be isolated within each intervention protocol.

PEDro and Cochrane risk-of-bias assessments were interpreted as complementary rather than interchangeable. PEDro scores summarized selected methodological and reporting features of the trials, whereas the Cochrane tools addressed the potential for bias in intervention-effect estimates. Accordingly, relatively higher PEDro scores should not be interpreted as evidence that a trial was necessarily at low risk of bias, and the PEDro categories were not used to modify the Cochrane judgments. At the study level, the descriptive RoB 1 domain summaries identified recurrent limitations, including incomplete reporting of allocation concealment, absence of participant and therapist blinding, variable assessor blinding, and lack of intention-to-treat analyses. However, these study-level RoB 1 domain summaries should not be interpreted as indicating that all outcomes within a trial were equally vulnerable to bias. Lack of participant or therapist blinding is frequently unavoidable in exercise-based interventions, and its potential impact depends on the type of outcome assessed. Self-reported outcomes, such as perceived ankle instability measured using the CAIT, may be more susceptible to expectation and reporting effects, whereas ultrasound-derived muscle morphology, instrumented kinematics, and other objectively quantified outcomes may be less directly influenced by participant awareness of group assignment. For the meta-analyzed dynamic postural control outcome, risk of bias was therefore evaluated separately using RoB 2 at the result level. The three contributing trials were classified as having some concerns overall rather than uniformly high risk of bias. These judgments reflected limitations specific to the reported dynamic postural control results, including incomplete reporting of some randomization or allocation procedures, aspects of outcome assessment, and the ability to verify selection of the reported result against prespecified analyses. Importantly, absence of participant blinding alone was not considered sufficient to determine high risk of bias. The outcome-specific RoB 2 judgments were subsequently incorporated into the GRADE assessment of the pooled evidence.

The principal evidence gap identified by this review should be distinguished from limitations of the review process itself. No eligible trial reported incident ankle injuries, prospectively recorded ankle-instability events, incident acute knee injuries, or incident knee-related symptoms/clinical syndromes; therefore, all predefined primary endpoint syntheses were empty. This represents a limitation of the available evidence base rather than a methodological limitation of the review. The review itself has several limitations that should be considered when interpreting its findings. First, quantitative synthesis was feasible for only one of the ten identified secondary outcome clusters (dynamic postural control; k = 3; n = 70), whereas the remaining outcomes required structured narrative synthesis. This limited the precision of the overall evidence synthesis and precluded formal exploration of potential effect modifiers such as exercise type, weekly dose, program duration, supervision, and participant clinical profile. Second, substantial clinical and measurement heterogeneity was present across the included studies. Populations ranged from healthy or recreationally active adults and runners to individuals with chronic ankle instability or flexible flatfoot, while outcome constructs were assessed using different instruments, operational definitions, and testing procedures. Even within the only pooled domain, dynamic postural control was assessed using related but non-interchangeable measures; therefore, standardization of effect sizes could address differences in scale but not differences in construct or measurement properties. Third, comparability across intervention protocols was limited. Training programs varied in exercise selection, progression, frequency, duration, supervision, and delivery format, with interventions ranging from approximately 4 to 10 weeks and including different combinations of short-foot, toe-flexion, toe-spread, doming, and other IFM-targeted exercises. Consequently, the present review cannot determine whether observed differences across studies reflect intervention efficacy, protocol-specific characteristics, population differences, or measurement variation. Fourth, the small number of studies contributing to individual outcome domains limited the robustness of pooled estimates and precluded reliable formal assessment of publication bias. Finally, quantitative inter-reviewer agreement statistics were unavailable for screening and risk-of-bias assessment because the archived review materials did not retain complete paired pre-consensus decisions required for valid retrospective κ estimation. These variables were therefore used only to characterize between-study variation and were not used to infer moderator effects.

An additional limitation concerns within-study outcome selection. Although a deterministic hierarchy was used to select a single result when studies reported multiple eligible measures within the same domain, the archived protocol did not retain a sufficiently granular prospective hierarchy covering all possible combinations of test direction, limb, follow-up time point, and balance metric. Residual risk of within-study outcome-selection bias therefore cannot be completely excluded. An additional methodological limitation is that formal RoB 2 assessment was restricted to the three dynamic postural-control results contributing to the meta-analysis. The sparse narrative outcome clusters were contextualized using descriptive RoB 1 domain summaries rather than formal result-specific RoB 2 judgments; consequently, risk-of-bias interpretation for those non-pooled outcomes remains qualitative.

A further limitation concerns the language restriction applied during study selection. Eligibility was limited to reports published in English or Portuguese because the review team did not have sufficient linguistic expertise or validated translation resources to ensure reliable assessment and data extraction in additional languages. Forty-seven records were excluded during screening because of an ineligible language or unavailable translation. This pragmatic restriction may have introduced language bias and reduced the comprehensiveness of the evidence base, particularly because intrinsic foot muscle research is conducted internationally, including in East Asian populations. Although several studies from Asian research settings were captured through English-language publications, relevant studies published exclusively in other languages may have been missed. The magnitude and direction of any resulting bias cannot be determined from the available data and should therefore be considered when interpreting the findings.

Generalizability is additionally constrained by the demographic and clinical composition of the included samples. Although the eligibility framework encompassed adults aged 18–59 years, most trials enrolled young adults, frequently university-aged, recreationally active, athletic, or otherwise biomechanically selected participants. Eleven of the 13 trials had group-level mean or median ages concentrated approximately between 19 and 30 years, and only two trials predominantly represented participants in their early forties [35,36]. Consequently, the findings should not be generalized without qualification to the broader adult population, particularly middle-aged, sedentary, or less physically active individuals, who were poorly represented or absent from the available randomized evidence. Population heterogeneity across healthy participants, runners, chronic ankle instability, and flexible flatfoot further limits the extent to which a single intervention effect can be assumed across clinical profiles.

Sex-specific interpretation is also limited by the reporting of the included trials. Although 11 studies enrolled participants of both sexes, outcome data were generally not reported separately for women and men, and two trials included men only [38,39]. Therefore, no sex-stratified quantitative or narrative synthesis could be performed. This limitation is particularly relevant because several lower-limb conditions discussed in the Introduction disproportionately affect women. The present findings should therefore not be assumed to represent equivalent intervention responses across sexes, and any implication for injury prevention in women should remain especially cautious. Future trials should ensure adequate representation of women and, where appropriately powered, report sex-stratified outcomes or formally test sex-by-treatment interactions.

The available surrogate evidence indicates that isolated IFM training may influence select intermediate foot-related outcomes, including strength, morphology, postural control, and perceived ankle instability. However, these findings should be interpreted as preliminary and hypothesis-generating rather than as evidence supporting clinical recommendations for injury prevention or broader rehabilitation effectiveness. None of the eligible randomized trials evaluated incident ankle injuries, prospectively recorded ankle-instability events, acute knee injury incidence, or incident knee-related symptoms/clinical syndromes. Accordingly, the available evidence does not establish that changes in these intermediate outcomes translate into clinically meaningful rehabilitation benefits or reduced injury risk. Among the interventions studied, short-foot exercise and related toe-extension and toe-spread variants were the most frequently investigated approaches; this frequency of investigation should not be interpreted as evidence of superior efficacy or as a basis for clinical recommendation.

In individuals with chronic ankle instability, flexible flatfoot, and running-specific populations, individual trials have reported changes in select foot- and ankle-related intermediate outcomes. However, these findings arise from small and heterogeneous evidence clusters, frequently involving single studies, and should not be interpreted as demonstrating reproducible treatment effects or supporting routine clinical implementation. At present, such findings primarily provide a rationale for further clinical investigation of isolated IFM training rather than a basis for recommendations regarding prevention or rehabilitation.

Critically, the absence of randomized trials measuring incident ankle injuries, prospectively recorded ankle-instability events, or either category of knee-related clinical endpoint means that any recommendation for primary injury prevention through isolated IFM training alone is currently not supported by direct empirical evidence and should be framed as biologically plausible but empirically untested. Future trials should prioritize: (i) larger, more homogeneous samples with adequate statistical power; (ii) standardization of outcome measures across studies; (iii) prospective assessment and separate reporting of incident ankle sprains, giving-way/ankle-instability events, acute knee injury events, and incident knee-related symptoms/clinical syndromes as predefined primary outcomes with adequate follow-up (minimum 6–12 months); (iv) inclusion of female participants with stratified analyses by sex; and (v) explicit reporting of allocation concealment, assessor blinding, and intention-to-treat analyses in accordance with CONSORT 2025 guidelines.

## 5. Conclusions

The available randomized evidence does not establish the effectiveness of IFM-targeted exercise programs for preventing ankle or knee injuries or for improving broader clinical outcomes. None of the included trials directly assessed the predefined primary clinical endpoints of incident ankle injury, prospectively recorded ankle-instability events, incident acute knee injury, or incident knee-related symptoms/clinical syndromes. Accordingly, the absence of eligible evidence for these endpoints should be interpreted as an unresolved evidence gap rather than as evidence of either benefit or no effect.

The available findings are preliminary and relate predominantly to intermediate biomechanical, neuromuscular, and functional outcomes. Dynamic postural control was the only secondary outcome amenable to quantitative synthesis, and the pooled estimate was non-significant, highly imprecise, and supported by Very-Low-certainty evidence. Other individual trials reported changes in foot muscle strength, morphology, posture, proprioception, perceived ankle instability, and inter-articular coordination; however, these findings were generally derived from small, heterogeneous studies and should not be interpreted as demonstrating established clinical effectiveness or injury-preventive benefit.

Therefore, the current evidence supports only the hypothesis that IFM-targeted exercise programs may influence select intermediate foot-related outcomes. Because some eligible programs incorporated ancillary foot- or ankle-focused exercises, observed effects should be interpreted at the level of the overall IFM-targeted intervention program and not as effects attributable exclusively to isolated intrinsic-foot-muscle contraction. Whether these adaptations translate into clinically meaningful improvements or reductions in ankle or knee injury incidence remains unknown. Appropriately powered prospective randomized trials incorporating predefined clinical endpoints and adequate follow-up are required before preventive or broader clinical effectiveness can be established.

## Figures and Tables

**Table 3:** Outcome-specific RoB 2 assessment for dynamic postural control.

Study	D1	D2	D3	D4	D5	Overall
Katsuya et al. [18]	Some concerns *	Low risk †	Low risk	Some concerns ‡	Some concerns §	Some concerns
Lee and Choi [19]	Some concerns *	Some concerns †	Low risk	Some concerns ‡	Some concerns §	Some concerns
Lynn et al. [20]	Some concerns *	Some concerns †	Low risk	Some concerns ‡	Some concerns §	Some concerns

D1: bias arising from the randomization process; D2: bias due to deviations from intended interventions; D3: bias due to missing outcome data; D4: bias in measurement of the outcome; D5: bias in selection of the reported result. * Random allocation was reported, but reporting of allocation concealment and/or the procedures used to generate and conceal the sequence was insufficient for an unequivocal low-risk judgment in all trials. † Participant blinding was inherently difficult in exercise interventions; judgments considered whether deviations from the assigned intervention attributable to the trial context were reported or likely to have materially affected the result. ‡ Dynamic postural control was assessed using established performance-based procedures; however, reporting of assessor blinding was incomplete in some trials. § Prospective protocols or sufficiently detailed prespecified statistical analysis plans allowing the selected dynamic postural control result to be verified against all eligible analyses were not consistently available.

**Table 5:** Structured narrative synthesis of non-pooled secondary outcomes according to SWiM guidance.

Outcome Cluster	Evidence Base	Direction and Magnitude of Effect	Consistency and Risk of Bias	Main Limitations
Foot muscle strength	4 randomized controlled trials 110 participants [30,34,36,39]	Generally favored intrinsec foot muscles training. Selected strength measures increased by approximately 13% to 37%, although comparable between-group effects were not consistently extractable.	Direction broadly consistent but transfer to higher-level function was not. Study-level concerns included incomplete allocation concealment, no participant/therapist blinding, and variable assessor blinding.	Different strength tests and reporting formats; small samples; surrogate outcome; uncertain clinical importance.
Foot muscle morphology	2 randomized controlled trials 66 participants [34,36]	Favored intrinsec foot muscles training. Increases in intrinsic foot muscle size/volume were reported using ultrasound.	Direction consistent. Objective imaging is less susceptible to expectation effects, but allocation and assessor-reporting limitations remain relevant.	Only two studies; different imaging methods and muscles; no direct link to injury incidence.
Foot posture	3 randomized controlled trials 78 participants [32,33,37]	Generally favored intrinsec foot muscles training. Reported changes were small, commonly about 0.5–1.5 points or 1–2 mm of navicular drop.	The direction was generally favorable across the small number of studies, but variation in measures, time points, and study populations limits any claim of reproducibility. Non-blinded exercise delivery and variable assessor blinding remain relevant.	Only three small trials; heterogeneous instruments, populations, and reporting; small absolute changes, some of which approach measurement error/minimal detectable change. Replication across comparable independent trials remains insufficient. Statistically significant changes should not be assumed to be clinically meaningful when their magnitude approaches measurement error or the minimal detectable change.
Static postural control/balance	3 randomized controlled trials 76 participants [18,32,39]	Mostly favored intrinsec foot muscles training in selected single-leg balance and center-of-pressure/center-of-gravity measures.	Direction generally favorable, but tasks and metrics differed. Performance-based measures reduce but do not eliminate assessor/expectation bias.	Different balance tasks and metrics; small samples; surrogate outcome.
Subjective ankle instability	1 randomized controlled trials 24 participants [18]	Favored intrinsec foot muscles training; Cumberland Ankle Instability Tool scores improved relative to control.	Consistency across studies cannot be assessed. Self-reported outcomes are particularly susceptible to expectation effects in an unblinded exercise trial.	Single small study; self-reported outcome; no injury-incidence follow-up.
Ankle kinesthesia/proprioception	1 randomized controlled trials 24 participants [39]	Favored intrinsec foot muscles training for selected sensorimotor measures.	Consistency cannot be assessed. Small-trial and non-blinding concerns remain relevant.	Single study; limited external validity; surrogate endpoint.
Inter-articular running coordination	2 randomized controlled trials; 127 participants [35,38]	Favored intrinsec foot muscles training mechanistically; both trials reported altered coupling/coordination patterns or reduced coordination variability.	Direction broadly consistent at a mechanistic level. Instrumented kinematics are relatively objective, but allocation and selective-reporting concerns remain.	Different coupling variables; mechanistic interpretation only; no prospective injury surveillance.
Running mechanics/running economy	2 randomized controlled trials 48 participants [30,36]	Mixed. One trial reported increased vertical propulsive impulse, whereas another found no meaningful change in joint mechanics or running economy despite strength gains.	Inconsistent across higher-level performance outcomes. Objective biomechanical measures reduce expectation bias, but small-sample limitations remain.	Different constructs and protocols; only two studies; no injury-incidence outcome.
Motor performance/perceived exercise difficulty	1 randomized controlled trial 23 participants [31]	Favoredintrinsec foot muscles training for toe-spread-out performance and perceived difficulty; ultrasound-derived activation did not show a clear between-group improvement.	Consistency cannot be assessed. Perceived difficulty is self-reported; small-sample and assessor-related concerns remain.	Single study; mixed objective/subjective measures; no direct clinical endpoint.

## Data Availability

The search strategy and Supplementary Materials are available at https://doi.org/10.17605/OSF.IO/TSMQG.

## Supplementary Materials

The following supporting information can be downloaded at https://www.mdpi.com/article/10.3390/sports14100455/s1, PRISMA 2020 Checklist.

## Abbreviations

The following abbreviations are used in this manuscript:

IFM	Intrinsic foot muscle
CAI	Chronic ankle instability
PFPS	Patellofemoral pain syndrome
